# Preserving joint line orientation in TKA improves short‐ to mid‐term outcomes: A systematic review and meta‐analysis

**DOI:** 10.1002/jeo2.70458

**Published:** 2025-10-31

**Authors:** Dúnio Jácome‐Pacheco, Tiago Torres, Gonçalo Rodrigues, Pedro Diniz, Francisco Guerra‐Pinto, António Camacho, João Gamelas, Romain Seil, Michael T. Hirschmann

**Affiliations:** ^1^ Hospital Ortopédico de Sant'Ana Lisbon Portugal; ^2^ NOVA Medical School Universidade NOVA de Lisboa Lisbon Portugal; ^3^ Luxembourg Institute of Research in Orthopaedics Sports Medicine and Science (LIROMS) Luxembourg Luxembourg; ^4^ Department of Bioengineering, iBB – Institute for Bioengineering and Biosciences, Instituto Superior Técnico Universidade de Lisboa Lisbon Portugal; ^5^ Centro de Responsabilidade Integrado de Traumatologia Ortopédica (CRI‐TO) do Centro Hospitalar Universitário de Lisboa Central (CHULC) Lisbon Portugal; ^6^ Direção Clínica Hospitalar da Unidade Local de Saúde de Lisboa Ocidental Lisbon Portugal; ^7^ Department of Orthopaedic Surgery and Traumatology Kantonsspital Baselland Bruderholz Switzerland

**Keywords:** joint line preservation, knee function, personalized arthroplasty, patient‐reported outcome measures, total knee arthroplasty

## Abstract

**Purpose:**

Joint line orientation (JLO) has been identified as a potential factor influencing clinical outcomes following total knee arthroplasty (TKA). This systematic review and meta‐analysis aimed to assess whether preserving the JLO according to the individual knee phenotype is associated with improved clinical and functional outcomes. We hypothesised that joint line preserving (JLP) techniques would result in superior patient‐reported outcome measures (PROMs) and better functional performance compared to non–joint line preserving (nJLP) approaches.

**Methods:**

A systematic search of Pubmed, CENTRAL, and Web of Science was conducted to identify comparative studies evaluating JLP versus nJLP in TKA. Studies reporting PROMs and other clinical indicators with a minimum follow‐up of 12 months were included. Risk of bias was assessed using the RoB 2 tool for randomised trials and the ROBINS‐I tool for non‐randomised studies. Meta‐analyses were performed for PROMs and range of motion, with subgroup analyses based on study quality.

**Results:**

Forty‐three studies were included in the qualitative analysis, and 38 in the meta‐analysis. The Forgotten Joint Score (MD: 7.59), Knee Function – Knee Society Score 2011 (MD: 6.48), Knee Injury and Osteoarthritis Outcome Score (MD: 2.74) and Oxford Knee Score (MD: 1.02) all showed statistically significant differences favouring JLP. Most subgroup analysis of low and low‐to‐moderate risk of bias studies further supported these effects.

**Conclusion:**

Joint line preservation in TKA is associated with short‐ to mid‐term improvements in PROMs and other clinical outcomes. While the effect may vary across patient populations, these findings support the relevance of JLO in optimising functional results. A more comprehensive and standardised phenotypic approach could be key to better identifying the subgroups that benefit most from this strategy.

**Level of Evidence:**

Level III.

## Graphical abstract

This systematic review and meta‐analysis found that preserving joint line orientation in total knee arthroplasty improves short‐ to mid‐term outcomes. Joint line–preserving techniques significantly enhanced PROMs like FJS, KOOS, OKS and Knee Society scores. Results suggest that aligning TKA to individual knee phenotypes may optimise function, supporting more personalised, phenotype‐based surgical strategies.

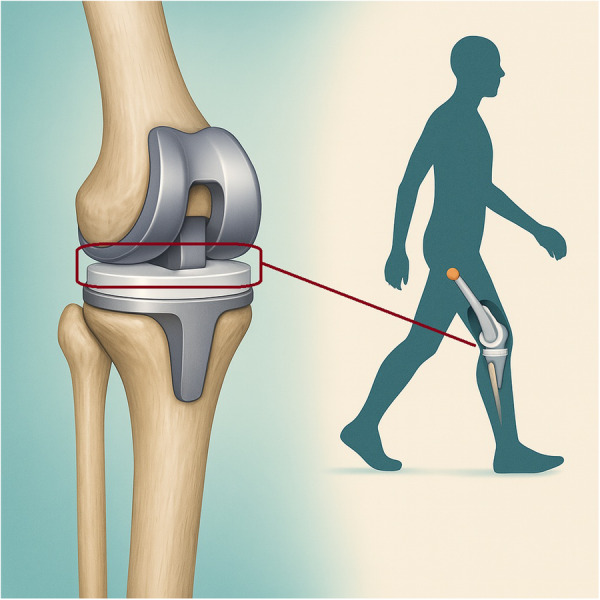

**Abbreviations**
ADL: activities of daily living
CI: confidence interval
CPAK: coronal plane alignment of the knee
EQ‐5D: EuroQol‐5 dimensions
FJS: Forgotten Joint Score
HKA: hip‐knee‐ankle angle
IQR: interquartile range
I²: I‐squared (statistical measure of heterogeneity)
JLO: joint line orientation
JLP: joint line‐preserving
KOOS: Knee Injury and Osteoarthritis Outcome Score
KOOS‐JR: KOOS joint replacement
KSS: Knee Society Score
MCID: minimum clinically important difference
MD: mean difference
MLN: method for unknown non‐normal distributions
nJLP: non‐joint line preserving
OKS: Oxford Knee Score
PACS: Picture Archiving and Communication System
PRISMA: Preferred Reporting Items for Systematic Reviews and Meta‐Analyses
PROMs: Patient‐Reported Outcome Measures
PROSPERO: International Prospective Register of Systematic Reviews
QE method: quantile estimation method
QoL: quality of life
RCT: randomised controlled trial
ROBINS‐I: Risk of Bias in Non‐randomised Studies ‐ of Interventions
RoB 2: Risk of Bias Tool for Randomised Trials
ROM: range of motion
SD: standard deviation
SF‐36: Short Form Health Survey 36
SPSS: IBM Statistical Package for the Social Sciences
TKA: total knee arthroplasty
VAS: visual analogue scale
WOMAC: Western Ontario and McMaster Universities Osteoarthritis Index

## INTRODUCTION

Total knee arthroplasty (TKA) remains associated with a significant dissatisfaction rate [15, 20], with a small proportion of patients reporting high levels of satisfaction [50], particularly among younger and more physically active individuals [35].

Suboptimal outcomes following total knee arthroplasty (TKA) are often linked to persistent pain, stiffness, patellofemoral complications, instability, infection and implant malposition [39, 69]. Additionally, patient‐related factors—including high preoperative pain levels, anxiety, comorbidities and unrealistic expectations—have also been implicated [15, 24, 46]. Among the biomechanical factors, growing attention has been directed toward the change in coronal joint line orientation (JLO) [3, 11, 28, 66, 67, 75, 79]. In the native knee, the JLO is naturally aligned parallel to the floor during gait, a configuration that facilitates stable joint mechanics, efficient load absorption and effective propulsion during the gait cycle [59]. Disruptions in this parallelism may impair functional outcomes, particularly during walking and other weight‐bearing activities.

The mechanical alignment philosophy, still the most widely adopted approach in TKA, often results in an oblique joint line orientation due to its strict perpendicularity to the mechanical axis of the limb. In contrast, emerging alignment strategies aim to more closely replicate native knee anatomy, with particular consideration for preserving joint line orientation [40, 78].

Preserving joint line orientation in total knee arthroplasty shows mixed evidence regarding its impact on short‐ to mid‐term outcomes [14, 19, 47, 65].

This systematic review aims to clarify whether the preservation of joint line orientation is associated with improved clinical outcomes in TKA. By examining the potential correlation between joint line preservation and patient‐reported outcomes, this study seeks to provide valuable insights into optimising alignment strategies to enhance patient satisfaction and overall success.

We hypothesised that joint line preserving techniques would result in superior patient‐reported outcome measures (PROMs) and better functional performance compared to non–joint line preserving approaches.

## MATERIALS AND METHODS

This systematic review was conducted according to the PRISMA (Preferred Reporting Items for Systematic Reviews and Meta‐Analyses) guidelines [44] and Cochrane recommendations [23] The protocol of this systematic review was registered on PROSPERO (CRD42023473589).

### Eligibility criteria

From title and abstract, all studies addressing joint line considerations in primary TKA were screened for inclusion. Exclusion criteria comprised studies involving cadavers, animals, or computer‐based models, as well as case reports, trial protocols, surgical technique descriptions, narrative reviews, and expert opinion articles. From full‐text review, only studies that compared joint line preserving (JLP) and non–joint line preserving (nJLP) alignment techniques were included, provided they reported PROMs. Classification into JLP or nJLP groups was determined based on the pre‐ and postoperative JLO imaging assessment and/or based on the surgical approach described. Eligible studies involved patients with degenerative knee pathology undergoing primary TKA, with no history of knee instability, infection, trauma, or deformity, and no prior surgeries affecting lower limb alignment. A minimum follow‐up of 12 months was required.

### Information sources

The database Pubmed, CENTRAL and Web of Science were searched for all published articles. No time restrictions were applied. Relevant references extracted from the articles screened were included as well. Searches were conducted on 16 February 2025.

### Search strategy

The following search string was used: ((knee replacement OR knee arthroplasty OR knee prosthesis) AND (joint line OR joint‐line) AND (prognosis OR prognostic OR prognostics OR outcomes OR outcome OR function OR patient‐reported outcome measures OR PROMs)).

### Study selection

Two reviewers (DJP and TT) independently screened the titles and abstracts yielded by the search against the inclusion criteria using the Rayyan QCRI systematic review management software [55]. Full reports for all titles and abstracts that met the inclusion criteria or when there was any uncertainty were collected. Full text reports were screened independently for inclusion and disagreements were settled by discussion or with a third‐party consultation (FGP).

### Data collection process and data items

Customised forms, Microsoft Excel, and Rayyan QCRI were used for data management. Two reviewers (DJP and TT) independently collected data, with extraction performed in duplicate. The following data items were collected: authors, year of publication, study type, level of evidence, participant eligibility and recruitment method, TKA alignment technique groups, joint line‐preserving assessment/method, knee prosthetic implant, robotic/navigation system utilisation, follow‐up period, preoperative population matching, outcome scales, outcome results and tested statistical correlations.

### Outcomes and prioritisation

The primary outcome measures were validated patient‐reported outcome measures (PROMs), including the Forgotten Joint Score (FJS), Oxford Knee Score (OKS), Knee Society Score (KSS), Western Ontario and McMaster Universities Osteoarthritis Index (WOMAC) and Knee Injury and Osteoarthritis Outcome Score (KOOS). Secondary outcomes included clinical indicators such as range of motion (ROM), pain assessed using the Visual Analogue Scale (VAS), health‐related quality of life (e.g., SF‐36 or EQ‐5D), kinematic and kinetic parameters from gait analysis.

### Study quality assessment

Quality assessment for interventional studies were done independently by two reviewers (DJP and TT) with the Cochrane Risk‐of‐bias Tool for Randomised Trials, RoB 2 [23], and with the Risk Of Bias In Non‐randomised Studies ‐ of Interventions, ROBINS‐I [23].

Besides the criteria mentioned by the tool itself, some specific issues were also considered. The “deviations from the intended intervention” domain in RoB 2 and the “classification of the intervention” domain in the ROBINS‐I tool were used to evaluate the accuracy of JLO preservation. The study quality was rated highest when detailed pre‐ and postoperative JLO assessments were included, with CT‐based JLO assessments receiving the highest scores, followed by medial proximal tibial angle (MPTA) measurements on full‐length lower‐limb radiographs. Additionally, or if these assessments were not fully available, JLO preservation quality was evaluated based on surgical alignment technique. Joint line‐preserving accuracy received higher scores when navigation or robotic assistance was utilised, CT/MRI‐based planning was incorporated, or an unrestricted, calipered‐measured kinematic alignment was performed. Disagreements were settled by discussion or with a third‐party consultation (FGP).

### Statistical analysis

Meta‐analyses of means and standard deviations (SDs) for PROMs and ROM were conducted using IBM SPSS Statistics (Version 29.0.0.0). All studies providing suitable data for quantitative analysis were included. In cases where multiple publications reported on the same patient cohort, the study with the longest follow‐up was selected to avoid data duplication—except when loss to follow‐up exceeded 85%, in which case the earlier study was included. Weighted mean differences (MDs) with 95% confidence intervals (CIs) were calculated for continuous variables. When only median and interquartile range (IQR) were reported, means and SDs were estimated using the Quantile Estimation (QE) method proposed by McGrath et al. [43]. For studies reporting median and range, the Method for Unknown Non‐Normal Distributions (MLN) developed by McGrath et al. [43] was used. In cases where only the mean and p‐value were available, SDs were calculated following Cochrane guidelines [23]. Whenever only the mean and range were reported, the SD was estimated using the method proposed by Wan et al. [76]. Detailed formulas are provided in the Supplementary File.

The overall KOOS score was calculated as the average of the KOOS subscales: Symptoms, Pain, Activities of Daily Living (ADL), Sport and Recreation (Sport/Rec), and Quality of Life (QoL). The overall standard deviation (SD) for the KOOS score was calculated by summing the squared SDs of the five subscales, dividing the total by five, and then taking the square root of the result. For the KOOS score, meta‐analysis was restricted to studies reporting the complete KOOS score. For KOOS‐JR, studies reporting KOOS domains closely aligned with KOOS‐JR—specifically Symptoms, Pain and ADL subscales—were included, while the Sport/Rec and QoL domains were excluded. Heterogeneity was assessed using the Q‐statistic, and no significant differences were found between subgroups (*Q* = 0.25, *df* = 1, *p* = 0.61; see Supporting Information: File S1). Accordingly, the subgroups were combined in a pooled meta‐analysis.

Knee objective indicators from KSS 1989 version [29] and KSS 2011 version [68] were pooled due to their similarity. Heterogeneity was assessed using the Q‐statistic, and between‐subgroup heterogeneity was not statistically significant (*Q* = 2.53, *df* = 1, *p* = 0.11; see Supporting Information: File S1), supporting their combination in a pooled meta‐analysis. In contrast, Function scores from the KSS 1989 and KSS 2011 versions were analysed separately, as the between‐subgroup heterogeneity was statistically significant (*Q* = 5.21, *df* = 1, *p* = 0.02; see Supporting Information: File S1), indicating meaningful differences between the two versions.

WOMAC global score was calculated as the average of its three domains: Pain, Stiffness, and Physical Function. The overall SD for the WOMAC score was determined by summing the squared SDs of the three subscales, dividing by three, and taking the square root of the result. For the WOMAC meta‐analysis, WOMAC scores reporting on a 0–100 “best” scale were converted to the original 0–100 “worst” scale to ensure consistency across studies.

For ROM, total ROM was calculated by adding the mean extension and flexion values. The total SD for ROM was derived by combining the variances of extension and flexion (squared SDs) and taking the square root of the sum.

For studies reporting both preoperative and postoperative PROMs, improvement scores were calculated, if already not provided. When specific information on the correlation between preoperative and postoperative scores was unavailable, a correlation value of 0.5 (*r* = 0.5) was assumed (Supporting Information: File S1 for details) [23].

Subgroup analyses were conducted based on study quality, focusing on studies assessed as having a low risk of bias. However, when fewer than five studies met this criterion—potentially compromising the reliability and interpretability of the meta‐analysis results [6] —studies with low to moderate risk of bias were included in the subgroup analyses. Due to the similarity in joint line preservation accuracy and overall study design within these subgroups, a fixed‐effect model was applied to enhance the precision of effect estimates. For the overall meta‐analysis, a random‐effects model was used to account for potential between‐study heterogeneity. Heterogeneity was assessed using the I² statistic, with values of 0%, 25%, 50% and 75% interpreted as no, low, moderate and high heterogeneity, respectively.

## RESULTS

**Figure 1:**
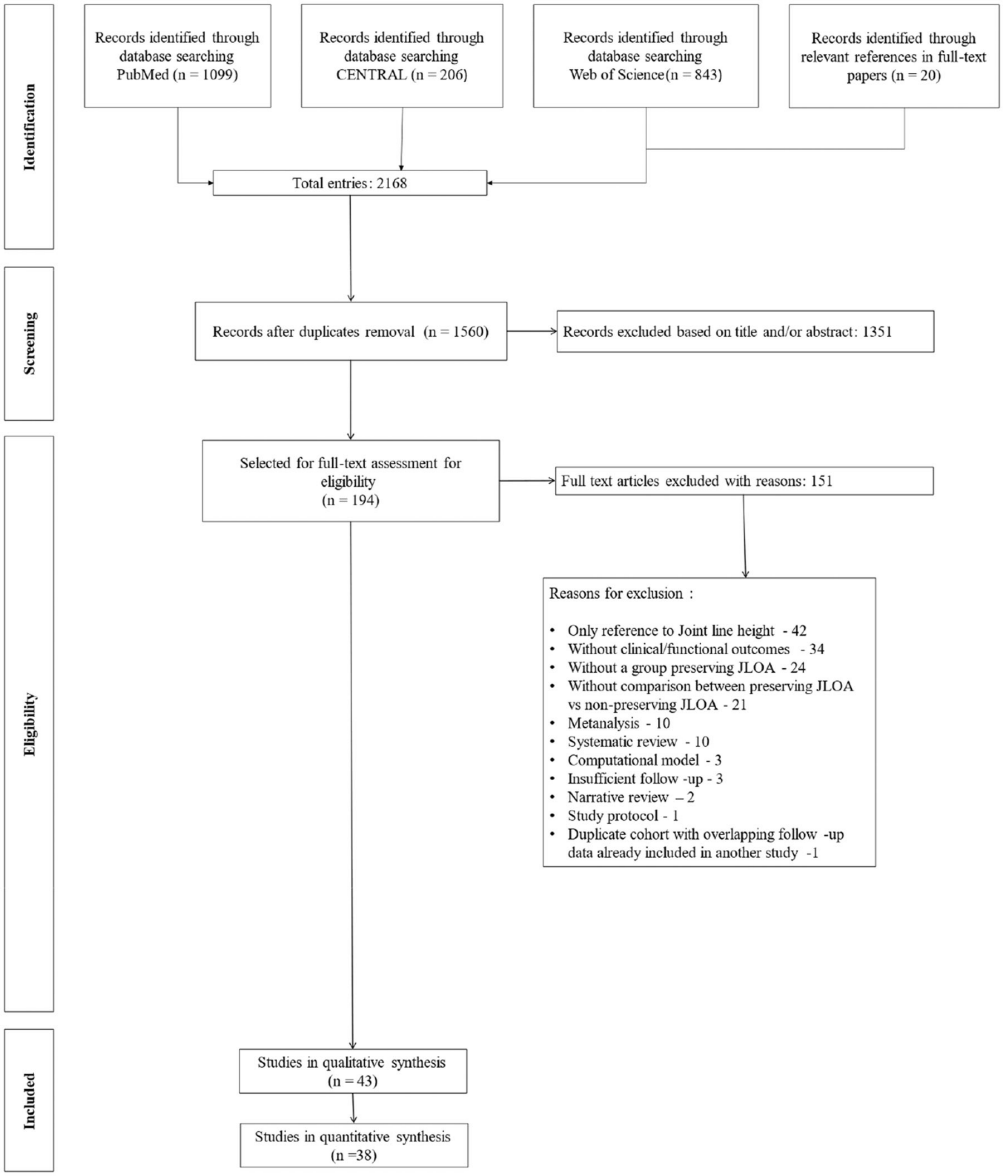
Study selection flow chart.

A total of 2168 records were identified. After removal of duplicate entries, 1560 studies underwent title and abstract screening. Based on predefined inclusion criteria, 194 studies were selected for full‐text review. Following further exclusions, 43 studies were included in the final qualitative synthesis. Study search and selection flowchart, with exclusion reasons, can be seen in Figure 1.

### General study characteristics

A total of 6357 TKA were analysed across all studies, of which 2715 were JLP procedures, while 3642 cases were nJLP procedures. Various alignment techniques were employed in JLP group, including kinematic alignment (KA), restricted kinematic alignment (rKA), functional alignment (FA) and adjusted mechanical alignment among other variants, while mechanical alignment (MA) was predominantly used in nJLP approaches.

Of the studies included in the qualitative analysis, 16 were randomised controlled trials (RCTs) evidence Level I [16, 22, 34, 37, 40, 41, 42, 48, 77, 80, 81, 82, 83] or II [9, 19, 31], seven were prospective cohort studies evidence Level II [11, 18, 51, 52, 53, 60, 79], 15 were retrospective cohort studies evidence Level III [4, 8, 10, 14, 32, 36, 47, 49, 56, 57, 61, 63, 65, 70, 72], and five were case–control studies evidence Level III [5, 30, 58, 71, 78]. Follow‐up ranged from 12 months to 10 years, with a mean follow‐up of approximately 36.4 months. Data related to individual study characteristics is further detailed in Table 1.

**Table 1:** General study characteristics.

Study	Type/level of evidence	Quality	JLP assessment/method	Alignment technique groups	Prosthesis/computer or robotic assisted	Number of TKA (JLP vs. nJLP)	Outcome scales	Outcomes Favouring JLP (Statistically Significant)
Dosset et al. [16]	RCT Level I	Some concerns^a^	MPTA	PSI KA vs. MA	Vanguard®/‐	44 vs. 44	KSS; OKS; Pain free %; ROM; WOMAC	KSS; OKS; Pain free %; ROM; WOMAC
Waterson et al. [77]	RCT Level I	Some concerns^a^	MRI‐based cutting guides; MPTA	PSI KA vs. MA	Triathlon®/‐	26 vs. 21	2 min walk; EQ‐5D; KOOS; KSS; Peak Tq hams; Peak Tq quads; ROM; SF‐36; TUG; UCLA	Peak Tq hams
Calliess et al. [9]	RCT Level II	Some concerns^a^	MRI‐based cutting guides; MPTA	PSI KA vs. MA	Triathlon®/‐	100 vs. 100	KSS; WOMAC	KSS; WOMAC
Matsumoto et al. [40]	RCT Level I	Some concerns^a^	JLO relative to the floor	rKA vs. MA	E‐motion® or Persona®/Navigation system Orthopilot 4.2	30 vs. 30	KSS; ROM	KSS
Niki et al. [51]	Prospective cohort Level II	Moderate risk of bias^b^	CT‐based measured resection	KA vs. MA	LPS‐FLEX®/ATHENA®	21 vs. 21	KAM; KSS; ROM	KAM
Young et al. [81]	RCT Level I	Low risk of bias^a^	MRI‐based cutting guides	PSI KA vs. MA	Triathlon®/PSI KA ‐ OtisMed®; MA computer navigation system Stryker®	49 vs. 50	EQ‐5D; FJS; KSS; OKS; ROM; VAS; WOMAC	‐
Rames et al. [61]	Retrospective cohort Level III	Moderate risk of bias^b^	MPTA	RV vs. MA	No mention/‐	50 vs. 181	FJS; OKS; SF‐12 MCS; SF‐12 PCS	‐
Nakajima et al. [49]	Retrospective cohort Level III	Serious risk of bias^b^	AJLMA	MA AJLMA ≥ 2° vs. MA AJLMA < 2°	FINE® 3° inclined PE/‐	74 vs. 75	KOOS; KSS	KOOS‐ADL
Murakami et al. [47]	Retrospective cohort Level III	Serious risk of bias^b^	MPTA	MA 3° PE vs. MA	Journey II® BCS; NexGen® PS/‐	23 vs. 23	KSS	KSS function
Niki et al. [52]	Prospective cohort Level II	Low risk of bias^b^	CT‐based measured resection	KA vs. MA	LPS‐FLEX®/ATHENA®; iASSIST® navigation system	45 vs. 45	KSS; ROM	KSS function
Blakeney et al. [5]	Case–control Level III	Moderate risk of bias^b^	JLO relative to the floor; MPTA	rKA vs. MA	Triathlon®/Orthomap® navigation	18 vs. 18	Kinematics analysis; KOOS	KOOS; Knee abduction–adduction curves; Swing phase knee flexion; Walking speed
Yeo et al. [80]	RCT Level I	High risk of bias^a^	Coronal tibial inclination	rKA vs. MA	NexGen®/ROBODOC®	30 vs. 30	Gait analysis; HSS; KSS; ROM; WOMAC	GRF medial–lateral
Laende et al. [34]	RCT Level I	Some concerns^a^	MRI‐based cutting guides; MPTA	PSI KA vs. MA	Triathlon®/PSI KA ‐ OtisMed®; CAS‐MA Precision Navigation System®	24 vs. 23	OKS; UCLA; VAS	‐
McEwen et al. [42]	RCT Level I	Some concerns^a^	JLO relative to the floor	rKA vs. MA (bilateral TKA)	Triathlon®/Precision Navigation System®	41 vs. 41	FJS; Knee preference; KOOS; KOOS‐JR; OKS; ROM	Knee preference
MacDessi et al. [37]	RCT Level I	Low risk of bias^a^	MPTA	rKA vs. MA	Legion®/Orthomap® navigation	70 vs. 68	EQ‐5D; FJS; KOOS	‐
Niki et al. [53]	Prospective cohort Level II	Low risk of bias^b^	CT‐based measured resection; MPTA	KA vs. MA	NexGen Zimmer‐Biomet®/OrthAlign® navigation	100 vs. 100	KSS; Pain catastrophizing scale; PDS; ROM	KSS function
Jeremić et al. [30]	Case‐control Level III	Moderate risk of bias^b^	MPTA	KA vs. MA	GMK Sphere®– Medacta/‐	24 vs. 24	FJS; KSS; KOOS	FJS; KOOS‐Sports; KSS
Young et al. [82]	RCT Level I	Low risk of bias^a^	MRI‐based cutting guides	PSI KA vs. MA	Triathlon®/PSI KA ‐ OtisMed®; MA computer navigation system Stryker®	47 vs. 48	EQ‐5D; FJS; KSS; OKS; VAS; WOMAC	‐
Matsumoto et al. [41]	RCT Level I	Some concerns^a^	CT‐based navigation assisted resection	rKA vs. MA	E‐motion®/ Navigation system Orthopilot 4.2	30 vs. 30	KSS; ROM	KSS function; KSS Knee Score; KSS satisfaction; ROM
Shelton et al. [70]	Retrospective cohort Level III	Moderate risk of bias^b^	Calipered resection	KA vs. MA	No mention/‐	78 vs. 78	FJS; OKS	FJS; OKS
D'Amato et al. [14]	Retrospective cohort Level III	Serious risk of bias^b^	JLO relative to the floor	MA PHE restored vs. MA PHE changed	Genesis II®/‐	KOOS 47 vs. 29 KSS 55 vs. 35	KOOS; KSS	KOOS‐Symptoms
Yaron et al. [4]	Retrospective cohort Level III	Low risk of bias^b^	MPTA	KA vs. MA	No mention/‐	38 vs. 38	FJS; ROM	FJS
Kaneda et al. [31]	RCT Level II	Some concerns^a^	MPTA	KA vs. MA	Evolution®/‐	8 vs. 5	Kinematic analysis; KSS	Flexion femoral external rotation; Medial pivoting
Shin et al. [71]	Case‐control Level III	Moderate risk of bias^b^	G‐Plafond	RV vs. MA	E‐motion®/ Navigation system Orthopilot 4.2	99 vs. 99	FJS; KSS; WOMAC	FJS; WOMAC
Calek et al. [8]	Retrospective cohort Level III	Serious risk of bias^b^	JLO relative to the floor	MA Horizontal JLO vs. MA Oblique JLO	Attune® or LCS®/ Computer‐navigated	36 vs. 40	FJS; ROM	‐
Kim et al. [32]	Retrospective cohort Level III	Low risk of bias^b^	MPTA; JLO relative to the floor	KA vs. MA	Persona®/‐	42 vs. 126	KSS; ROM; SF‐36; WOMAC	‐
Sappey‑Marinier et al. [65]	Retrospective cohort Level III	Serious risk of bias^b^	MPTA + LDFA (joint line obliquity)	MA JLO restored vs. MA JLO changed	No mention/‐	194 vs. 884	KSS; ROM	PHE JLO < 177°: KSS Knee score; KSS Pain subscale
Cherches et al. [10]	Retrospective cohort Level III	Serious risk of bias^b^	MPTA	KA vs. MA	No mention/‐	95 vs. 35	KOOS JR; PROMIS; VR12	‐
Elbuluk et al. [18]	Prospective cohort Level II	Moderate risk of bias^b^	CT‐based robotic resection	KA vs. MA	No mention/MAKO Stryker® robotic‐assistance	100 vs. 100	FJS; KOOS JR; ROM; VAS; VR12	FJS; KOOS JR; VAS
Parratte et al. [58]	Case‐control Level III	Moderate risk of bias^b^	MPTA; JLO relative to the floor	AFIP vs. aMA	Persona®/rKA robotic‐assisted	40 vs. 40	KSS; ROM	KSS function
Clark et al. [11]	Prospective cohort Level II	Low risk of bias^b^	MPTA + LDFA (joint line obliquity)	FA(k) vs. FA(m)	Triathlon®/Mako® robotic system	165 vs. 135	EQ‐5D; FJS; KOOS JR; OKS; ROM; VAS	FJS; ROM
Yamada et al. [79]	Prospective cohort Level II	Serious risk of bias^b^	JLO relative to the floor	MA (3° PE) JLO restored vs. MA (3° PE) JLO changed	FINE®/‐	80 vs. 65	KOOS; KSS; ROM	ROM
Rak et al. [60]	Prospective cohort Level II	Moderate risk of bias^b^	TMA	MA PHE restored vs. MA PHE change	Triathlon®/‐	45 vs. 14	FJS; OKS; WOMAC	‐
Grave et al. [78]	Case‐control Level III	Low risk of bias^b^	MPTA	iKA vs. aMA	Triathlon®/Mako® robotic system	19 vs. 17	FJS; Gait analysis; OKS; ROM; VAS	FJS; Kinematic knee–sagittal ROM; OKS; ROM; VAS
Zheng et al. [83]	RCT Level I	Some concerns^a^	MPTA + LDFA (joint line obliquity)	CA vs. MA	Columbus®/Navigation system Orthopilot 5.1	38 vs. 38	FJS; HSS; ROM; WOMAC	‐
Stoltz et al. [72]	Retrospective cohort Level III	Serious risk of bias^b^	CT‐based robotic resection	RA‐FA vs. MA	Triathlon®/Not specified	393 vs. 312	FJS; KOOS‐JR; KSS; Likert scale; ROM; Satisfaction Rate; WOMAC	KOOS‐JR; KSS; Likert scale; ROM; Satisfaction rate; WOMAC
Pangaud et al. [56]	Retrospective cohort Level III	Moderate risk of bias^b^	MPTA + LDFA (joint line obliquity)	MA JLO restored vs. MA JLO changed	Persona®; Anatomic®/‐	26 vs. 152	FJS; KOOS; SKV	KOOS‐ADL; KOOS‐QoL
Lee et al. [36]	Retrospective cohort Level III	Low risk of bias^b^	MPTA + LDFA (joint line obliquity)	FA vs. MA	Triathlon®/MAKO Stryker® robotic‐assistance	70 vs. 140	FJS; KSS; VAS; WOMAC	FJS; KSS function; WOMAC
Ettinger et al. [19]	RCT Level II	Low risk of bias^a^	CT‐based measured resection; MPTA	PSI rKA VS PSI MA	GMK Sphere®/‐	47 vs. 51	FJS; KSS; OKS; WOMAC	FJS; KSS expectation; KSS function; KSS satisfaction
Park et al. [57]	Retrospective cohort Level III	Moderate risk of bias^b^	MPTA + LDFA (joint line obliquity)	MA JLO restored vs. MA JLO changed	Journey II® BCS; Persona® PS/‐	106 vs. 106	American KSS; Femoral Roll Back; FJS; ROM; WOMAC	Femoral roll back; FJS; ROM
Nakagawa et al. [48]	RCT Level I	Some concerns^a^	MPTA; JLO relative to the floor	AA vs. MA	Actiyas®/‐	40 vs. 40	KOOS‐Pain; KOOS‐Symptom; KS‐KSS; KSQ; Knee preference; ROM	Knee preference; KSQ – Satisfaction
Rodríguez et al. [63]	Retrospective cohort Level III	Moderate risk of bias^b^	MPTA + LDFA (joint line obliquity)	MA JLO restored vs. MA JLO changed	Saiph®/‐	16 vs. 78	FJS; KOOS; OKS; UCLA; VAS	‐
Gibbons et al. [22]	RCT Level I	Low risk of bias^a^	MRI‐based cutting guides	PSI KA vs. MA	Triathlon®/‐	39 vs. 42	EQ‐5D; FJS; KSS; OKS; WOMAC	‐

Abbreviations: AA, anatomic alignment; ADL, activities of daily living; AFIP, anatomo‐functional implant positioning; AJLMA, angle between the joint line and the line perpendicular to the mechanical axis; aMA, adjusted mechanical alignment; CA, constitutional alignment; CAS, computer‐assisted surgery; CT, computer tomography; EQ‐5D, EuroQol‐5 Dimension; FA(k), functional alignment with a kinematic alignment plan; FA(m), functional alignment with a mechanical alignment plan; FJS, Forgotten Joint Score; GRF, ground reaction force; G‐Plafond, tibial plafond orientation relative to the ground; Hams, hamstrings; HSS, Hospital for Special Surgery Knee Score; iKA, inverse kinematic alignment; JLO, joint line orientation; JLP, Joint Line Preserving; KA, kinematic alignment; KAM, Knee Adduction Moment; KOOS, Knee Injury and Osteoarthritis Outcome Score; KOOS JR, Knee Injury and Osteoarthritis Outcome Score for Joint Replacement; KS, Knee score; KSQ, Knee Society Questionnaire; KSS, Knee Society Score; LDFA, lateral distal femoral angle; MA, mechanical alignment; MPTA, medial proximal tibial angle; MRI, magnetic resonance imaging; nJLP, non‐Joint Line Preserving; OKS, Oxford Knee Score; PDS, pain DETECT score; PE, polyethylene; PHE, phenotype; PROMIS, Patient‐Reported Outcomes Measurement Information System; PSI, patient‐specific instrumentation; QoL, quality of life; Quads, quadriceps; RA‐FA, robotic‐assisted functional alignment; RCT, randomised control trial; rKA, restricted kinematic alignment; ROM, range of motion; RV, residual varus; SF‐12 MCS, 12‐Item Short Form Health Survey Mental Component Score; SF‐12 PCS, 12‐Item Short Form Health Survey Physical Component Score; SF‐36, Short Form‐36 Health Survey; SKV, subjective knee value; TKA, total knee arthroplasty; TMA, tibial mechanical angle; Tq, torque; TUG, Timed Up and Go; UCLA, University of California at Los Angeles activity score; VAS, Visual Analogue Scale; VASS, Visual Analogue Scale for Satisfaction; VR12, Veterans RAND 12‐Item Health Survey; WOMAC, Western Ontario and McMaster Universities Arthritis Index.

^a^ RoB 2.0: Revised Cochrane risk of bias tool version 2.0.

^b^ ROBINS‐I: Risk of bias in non‐randomised studies‐ of Interventions.

### Risk of bias

The overall risk of bias varied across study designs. Among the RCTs, based on the Cochrane RoB 2.0 tool, one study was classified as having a high risk of bias [80], ten had some concerns [9, 16, 31, 34, 40, 41, 42, 48, 77, 83] and five were rated as low risk of bias [19, 22, 37, 81, 82]. For non‐randomised studies, assessed using the Cochrane ROBINS‐I tool, eight studies were classified as having a serious risk of bias [8, 10, 14, 47, 49, 65, 72, 79], 12 had a moderate risk [5, 18, 30, 51, 56, 57, 58, 60, 61, 63, 70, 71] and seven were rated as low risk of bias [4, 11, 32, 36, 52, 53, 78]. The risk of bias in individual studies is shown in Tables 2.1 and 2.2.

**Table 2.1:** Risk of bias in randomised controlled trial studies.

Authors	Year	Randomisation process	Deviations from the intended interventions	Missing outcome data	Measurement of the outcome	Selection of the reported result	Overall
**RoB 2.0**							
Dosset et al. [16]	2014	Low risk	Some concerns	Low risk	Low risk	Low risk	Some concerns
Waterson et al. [77]	2016	Low risk	Some concerns	Low risk	Low risk	Low risk	Some concerns
Calliess et al. [9]	2017	Some concerns	Some concerns	Low risk	Some concerns	Low risk	Some concerns
Matsumoto et al. [40]	2017	Low risk	Some concerns	Low risk	Low risk	Low risk	Some concerns
Young et al. [81]	2017	Low risk	Low risk	Low risk	Low risk	Low risk	Low risk
Laende et al. [34]	2019	Low risk	Some concerns	Low risk	Low risk	Low risk	Some concerns
Yeo et al. [80]	2019	Low risk	High risk	Low risk	Low risk	Low risk	High risk
Matsumoto et al. [41]	2020	Low risk	Some concerns	Low risk	Low risk	Low risk	Some concerns
MacDessi et al. [37]	2020	Low risk	Low risk	Low risk	Low risk	Low risk	Low risk
McEwen et al. [42]	2020	Low risk	Some concerns	Low risk	Low risk	Low risk	Some concerns
Young et al. [82]	2020	Low risk	Low risk	Low risk	Low risk	Low risk	Low risk
Kaneda et al. [31]	2022	Some concerns	Some concerns	Low risk	Low risk	Low risk	Some concerns
Zheng et al. [83]	2024	Low risk	Some concerns	Low risk	Low risk	Low risk	Some concerns
Ettinger et al. [19]	2024	Low risk	Low risk	Low risk	Low risk	Low risk	Low risk
Nakagawa et al. [48]	2025	Low risk	Some concerns	Low risk	Low risk	Low risk	Some concerns
Gibbons et al. [22]	2025	Low risk	Low risk	Low risk	Low risk	Low risk	Low risk

**Table 2.2:** Risk of bias in non‐randomised studies.

Authors	Year	Confounding	Selection of participants into the study	Classification of interventions	Deviations from intended interventions	Missing data	Selection of the reported result	Overall
**ROBINS‐I**								
Niki et al. [51]	2017	Low risk	Low risk	Moderate risk	Low risk	Low risk	Low risk	Moderate risk
Nakajima et al. [49]	2018	Serious risk	Low risk	Serious risk	Low risk	Low risk	Low Risk	Serious risk
Rames et al. [61]	2018	Moderate risk	Low risk	Moderate risk	Low risk	Low risk	Low Risk	Moderate risk
Murakami et al. [47]	2018	Serious risk	Moderate risk	Serious risk	Low risk	Low risk	Low Risk	Serious risk
Niki et al. [52]	2018	Low risk	Low risk	Low risk	Low risk	Low risk	Low Risk	Low risk
Blakeney et al. [5]	2019	Low risk	Moderate risk	Low risk	Low risk	Low risk	Low Risk	Moderate risk
Jeremić et al. [30]	2020	Moderate risk	Low risk	Moderate risk	Low risk	Low risk	Low Risk	Moderate risk
Niki et al. [53]	2020	Low risk	Low risk	Low risk	Low risk	Low risk	Low risk	Low risk
Yaron et al. [4]	2021	Low risk	Low risk	Low risk	Low risk	Low risk	Low Risk	Low risk
D'Amato et al. [14]	2021	Serious risk	Low risk	Serious risk	Low risk	Low risk	Low Risk	Serious risk
Shelton et al. [70]	2021	Low risk	Moderate risk	Moderate risk	Low risk	Low risk	Low Risk	Moderate risk
Calek et al. [8]	2022	Low risk	Low risk	Serious risk	Low risk	Low risk	Low Risk	Serious risk
Cherches et al. [10]	2022	Serious risk	Low risk	Moderate risk	Low risk	Low risk	Low Risk	Serious risk
Kim et al. [32]	2022	Low risk	Low risk	Low risk	Low risk	Low risk	Low Risk	Low risk
Yamada et al. [79]	2023	Serious risk	Low risk	Serious risk	Low risk	Low risk	Low Risk	Serious risk
Shin et al. [71]	2022	Low risk	Low risk	Moderate risk	Low risk	Low risk	Low Risk	Moderate risk
Sappey‑Marinier et al. [65]	2022	Serious risk	Low risk	Serious risk	Low risk	Low risk	Low Risk	Serious risk
Elbuluk et al. [18]	2022	Low risk	Low risk	Moderate risk	Low risk	Low risk	Low Risk	Moderate risk
Parratte et al. [58]	2023	Moderate risk	Low risk	Moderate risk	Low risk	Low risk	Low Risk	Moderate risk
Rak et al. [60]	2023	Moderate risk	Low risk	Moderate risk	Low risk	Low risk	Low Risk	Moderate risk
Grave et al. [78]	2023	Low risk	Low risk	Low risk	Low risk	Low risk	Low Risk	Low risk
Clark et al. [11]	2023	Low risk	Low risk	Low risk	Low risk	Low risk	Low Risk	Low risk
Stoltz et al. [72]	2024	Serious risk	Moderate risk	Moderate risk	Low risk	Low risk	Low risk	Serious risk
Pangaud et al. [56]	2024	Moderate risk	Low risk	Low risk	Low risk	Low risk	Low risk	Moderate risk
Lee et al. [36]	2024	Low risk	Low risk	Low risk	Low risk	Low risk	Low risk	Low risk
Park et al. [57]	2025	Moderate risk	Low risk	Moderate risk	Low risk	Low risk	Low risk	Moderate risk
Rodríguez et al. [63]	2025	Moderate risk	Low risk	Low risk	Low risk	Low risk	Low risk	Moderate risk

### Findings

The included studies utilised a diverse range of outcome measures. The most consistently reported outcomes were KSS, reported in 27 studies [9, 14, 16, 19, 22, 30, 31, 32, 36, 40, 41, 47, 48, 49, 51, 52, 53, 57, 58, 65, 71, 72, 77, 79, 80, 81, 82], followed by FJS in 22 studies [4, 8, 11, 18, 19, 22, 30, 36, 37, 42, 56, 57, 60, 61, 63, 70, 71, 72, 78, 81, 82, 83], WOMAC in 14 studies [9, 16, 19, 22, 32, 36, 57, 60, 71, 72, 80, 81, 82, 83], OKS in 12 studies [11, 16, 19, 22, 34, 60, 61, 63, 70, 78, 81, 82], KOOS in 11 studies [5, 14, 30, 42, 48, 49, 56, 63, 65, 77, 79], KOOS‐JR in five studies [10, 11, 18, 42, 72] and VAS in eight [11, 18, 34, 36, 63, 78, 81, 82]. Additionally, objective assessments included ROM, reported in 23 studies [4, 8, 11, 16, 18, 32, 40, 41, 42, 48, 51, 52, 53, 57, 58, 65, 72, 77, 78, 79, 80, 81, 83], and various kinematic and kinetic parameters in seven studies [5, 31, 51, 57, 77, 78, 80], capturing functional and biomechanical outcomes.

Further details on outcome findings can be found in Table 1.

### Quantitative analysis

Among all papers, 38 were suitable for a quantitative meta‐analysis.

**Figure 2:**
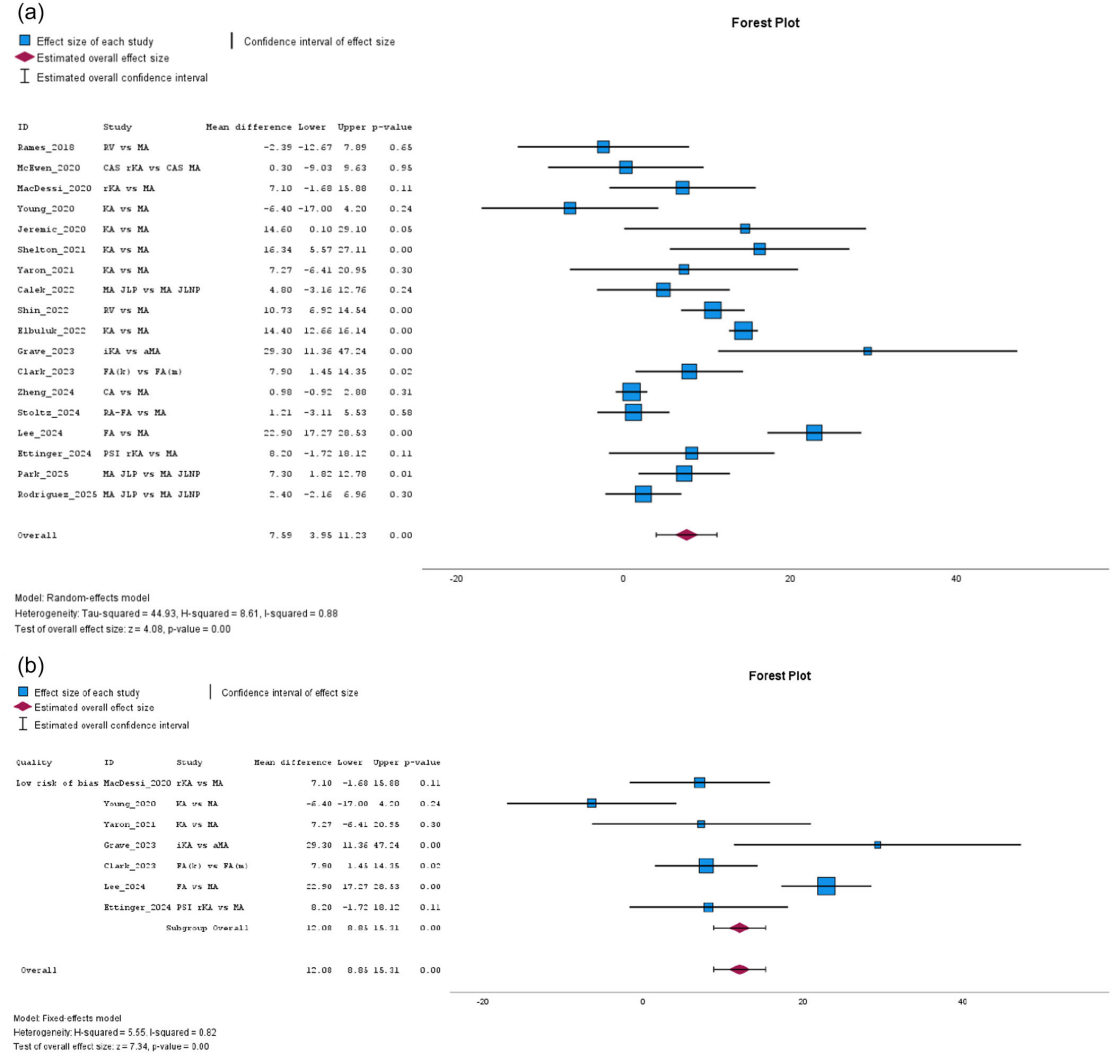
(a) Meta‐analysis forest plot for the Forgotten Joint Score (FJS). (b) Low risk of bias subgroup meta‐analysis for the FJS.

The FJS, from 18 studies [4, 8, 11, 18, 19, 30, 36, 37, 42, 57, 61, 63, 70, 71, 72, 78, 82, 83], significantly favoured the JLP group with high heterogeneity (MD: 7.59, 95% CI: 3.95–11.23, *p* = 0.00, *I*² = 88%, see Figure 2a). Subgroup analysis reinforced this effect in low risk of bias studies, with no heterogeneity (MD: 12.08, 95% CI: 8.85–15.31, *p* = 0.00, *I*² = 0%, *I*² = 0%, see Figure 2b). FJS improvement, analysed in four studies [8, 11, 37, 81], also favoured JLP, with no heterogeneity (MD: 4.72, 95% CI: 0.83–8.61, *p* = 0.02, *I*² = 0%), a finding further supported in subgroup analysis (MD: 5.69, 95% CI: 1.16–10.22, *p* = 0.01, *I*² = 0%).

The Knee Objective Score‐KSS, from 17 studies [14, 16, 19, 30, 32, 36, 41, 48, 49, 51, 57, 58, 65, 71, 72, 79, 82], showed no significant difference between groups, with high heterogeneity (MD: 1.09, 95% CI: –0.29 to 2.48, *p* = 0.12, *I*² = 80%). Subgroup analysis in low‐to‐moderate risk of bias studies also found no significant effect, with moderate heterogeneity (MD: 0.45, 95% CI: –0.29 to 1.20, *p* = 0.23, *I*² = 66%). Knee Objective Score‐KSS improvement, assessed in 13 studies [16, 19, 30, 32, 36, 41, 48, 51, 57, 58, 72, 79, 81], remained non‐significant (MD: 0.85, 95% CI: –1.15 to 2.84, *p* = 0.40, *I*² = 67%), as did the low‐to‐moderate risk of bias subgroup analysis (MD: –0.36, 95% CI: –1.60 to 0.88, *p* = 0.57, *I*² = 61%), both showing moderate heterogeneity.

**Figure 3:**
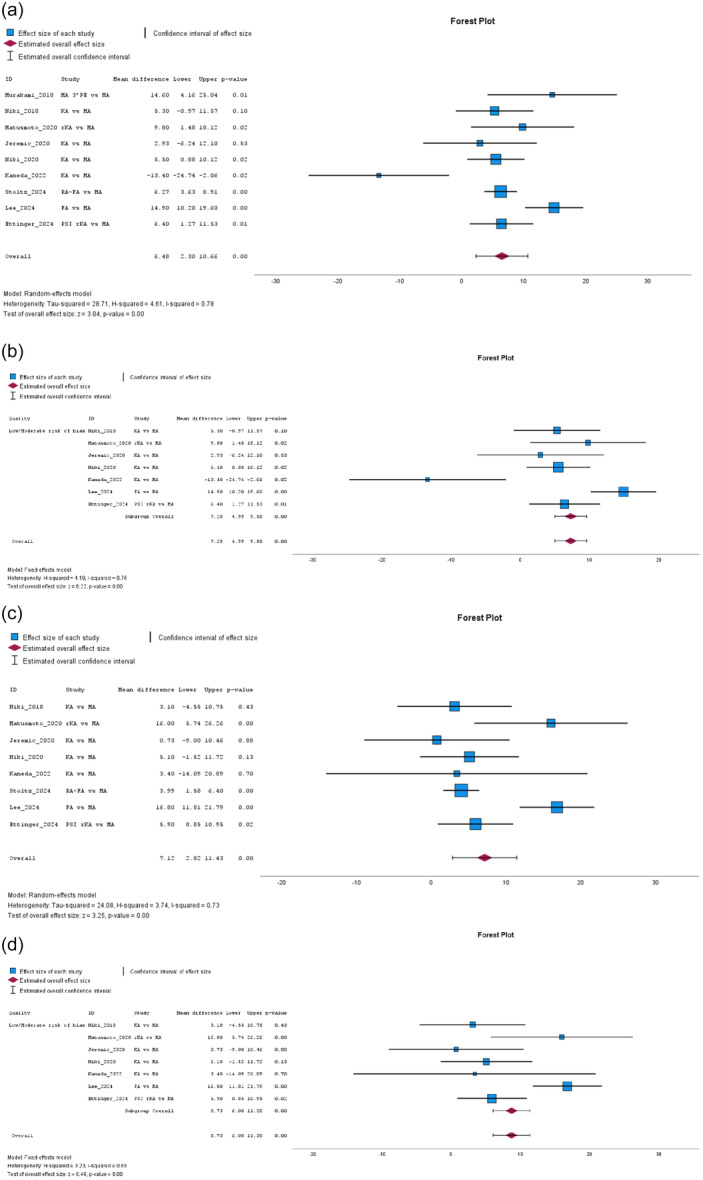
(a) Meta‐analysis forest plot for the Knee Function‐Knee Society Score (KSS) 2011. (b) Low‐to‐moderate risk of bias subgroup meta‐analysis for the Knee Function‐KSS 2011. (c) Meta‐analysis forest plot for the Knee Function‐KSS 2011 improvement. (d) Low‐to‐moderate risk of bias subgroup meta‐analysis for the Knee Function‐KSS 2011 improvement.

The Function score – KSS 1989 version [29] from 12 studies [14, 16, 32, 49, 57, 58, 65, 71, 77, 79, 80, 82] did not significantly favour JLP, with moderate heterogeneity (MD: 0.72, 95% CI: –1.92 to 3.36, *p* = 0.59, *I*² = 72%). Subgroup analysis in low‐to‐moderate risk of bias studies did not reach statistical significance, with moderate heterogeneity (MD: 1.00, 95% CI: −0.56 to 2.55, *p* = 0.21, *I*² = 58%). In contrast, the Function score – KSS 2011 version [68] from nine studies [19, 30, 31, 36, 41, 47, 52, 53, 72] significantly favoured the JLP group, with high heterogeneity (MD: 6.48, 95% CI: 2.30–10.66, *p* = 0.00, *I*² = 78%, see Figure 3a). Subgroup analysis in low‐to‐moderate risk of bias studies confirmed a significant effect, with high heterogeneity (MD: 7.28, 95% CI: 4.99–9.58, *p* = 0.00, *I*² = 76%, see Figure 3b). Improvement in Knee Function – KSS 1989 version, from seven studies [16, 32, 57, 58, 79, 80, 81], did not reach statistical significance (MD: 3.15, 95% CI: −1.54 to 7.84, *p* = 0.19, *I*² = 81%), with low‐to‐moderate risk subgroup analysis yielding as well non‐significant results (MD: 0.31, 95% CI: −1.71 to 2.33, *p* = 0.77, *I*² = 81%), both showing high heterogeneity. Improvement in Knee Function – KSS 2011 version, from eight studies [19, 30, 31, 36, 41, 52, 53, 72], significantly favoured JLP (MD: 7.12, 95% CI: 2.82–11.43, *p* = 0.00, *I*² = 73%, see Figure 3c), with low‐to‐moderate risk subgroup analysis reinforcing the effect (MD: 8.73, 95% CI: 6.08–11.38, *p* = 0.00, *I*² = 69%, see Figure 3d), both showing moderate heterogeneity.

**Figure 4:**
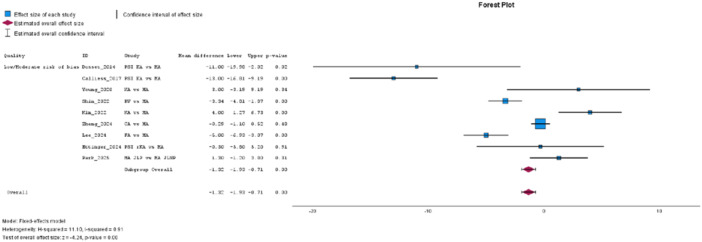
Low‐to‐moderate risk of bias subgroup meta‐analysis for the Western Ontario and McMaster Universities Osteoarthritis Index (WOMAC).

The WOMAC, from 11 studies [9, 16, 19, 32, 36, 57, 71, 72, 80, 82, 83], did not show a statistically significant difference between groups, with high heterogeneity (MD: −2.64, 95% CI: −5.61 to 0.34, *p* = 0.08, *I*² = 96%). Subgroup analysis of low‐to‐moderate risk of bias studies demonstrated a statistically significant difference favouring JLP, also with high heterogeneity (MD: −1.32, 95% CI: −1.93 to −0.71, *p* = 0.00, *I*² = 91%, see Figure 4). Improvement in WOMAC, analysed across 11 studies [9, 16, 19, 32, 36, 57, 71, 72, 80, 81, 83], was not statistically significant (MD: 1.23, 95% CI: −1.81 to 4.28, *p* = 0.43, *I*² = 86%), as was the subgroup analysis of low‐to‐moderate risk studies (MD: 0.76, 95% CI: −0.46 to 1.97, *p* = 0.22, *I*² = 87%), both revealing high heterogeneity.

**Figure 5:**
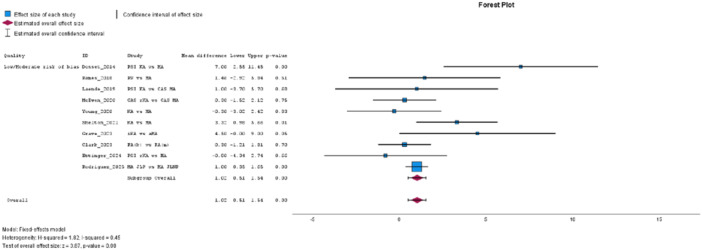
Meta‐analysis forest plot for the Oxford Knee Score (OKS).

The Oxford Knee Score (OKS), from 10 studies [11, 16, 19, 34, 42, 61, 63, 70, 78, 82]—all classified as low‐to‐moderate risk of bias—significantly favoured JLP, with low heterogeneity (MD: 1.02, 95% CI: 0.51–1.54, *p* = 0.00, *I*² = 45%, see Figure 5). Improvement in OKS, analysed in eight studies [11, 16, 19, 34, 42, 61, 78, 81]—all also classified as low‐to‐moderate risk—showed a borderline non‐significant pooled mean difference, with low heterogeneity (MD: 0.99, 95% CI: −0.06 to 2.04, *p* = 0.07, *I*² = 34%).

**Figure 6:**
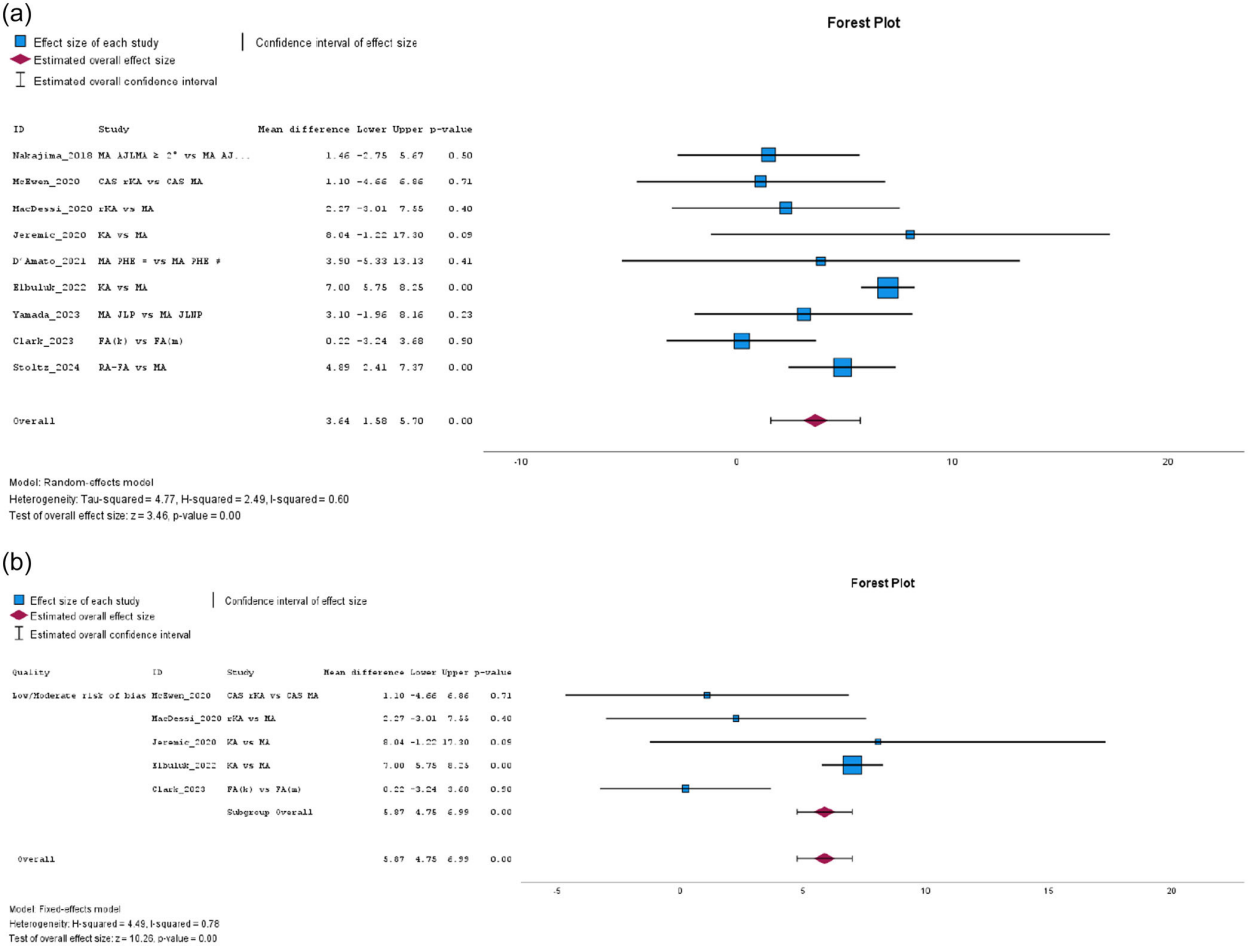
(a) Meta‐analysis forest plot for the Knee Injury and Osteoarthritis Outcome Score‐Joint Replacement (KOOS‐JR). (b) Low‐to‐moderate risk of bias subgroup meta‐analysis for the KOOS‐JR.

The KOOS JR, from nine studies [11, 14, 18, 30, 37, 42, 49, 72, 79], significantly favoured JLP, with moderate heterogeneity (MD: 3.64, 95% CI: 1.58–5.70, *p* = 0.00, *I*² = 60%, see Figure 6a). Subgroup analysis confirmed significance in low‐to‐moderate risk of bias studies, with high heterogeneity (MD: 5.87, 95% CI: 4.75–6.99, *p* = 0.00, *I*² = 78%, see Figure 6b). KOOS JR improvement, analysed in five studies [11, 30, 37, 42, 79], showed no statistically significant difference, with no heterogeneity (MD: 1.45, 95% CI: −0.89 to 3.78, *p* = 0.23, *I*² = 0%), consistent with low‐to‐moderate risk subgroup analysis (MD: 1.27, 95% CI: −1.36 to 3.90, *p* = 0.34, *I*² = 0%).

**Figure 7:**
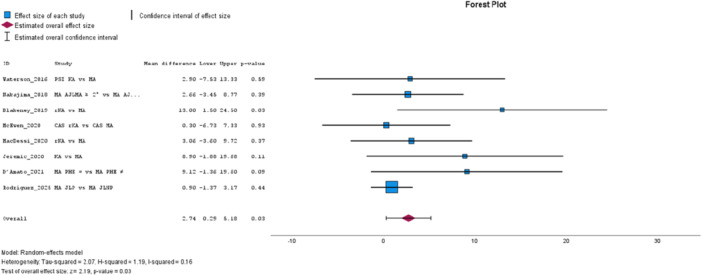
Meta‐analysis forest plot for the Knee Injury and Osteoarthritis Outcome Score (KOOS).

The KOOS, from eight studies [5, 14, 30, 37, 42, 49, 63, 77], significantly favoured JLP, with low heterogeneity (MD: 2.74, 95% CI: 0.29–5.18, *p* = 0.03, *I*² = 16%, see Figure 7). Subgroup analysis showed a borderline non‐significant effect in low‐to‐moderate risk of bias studies, with low heterogeneity (MD: 1.72, 95% CI: −0.23 to 3.67, *p* = 0.08, *I*² = 20%). Improvement in KOOS, analysed in four studies [5, 30, 37, 42]—all classified as low‐to‐moderate risk of bias—was borderline non‐significant, with no heterogeneity (MD: 3.87, 95% CI: −0.37 to 8.12, *p* = 0.07, *I*² = 0%).

**Figure 8:**
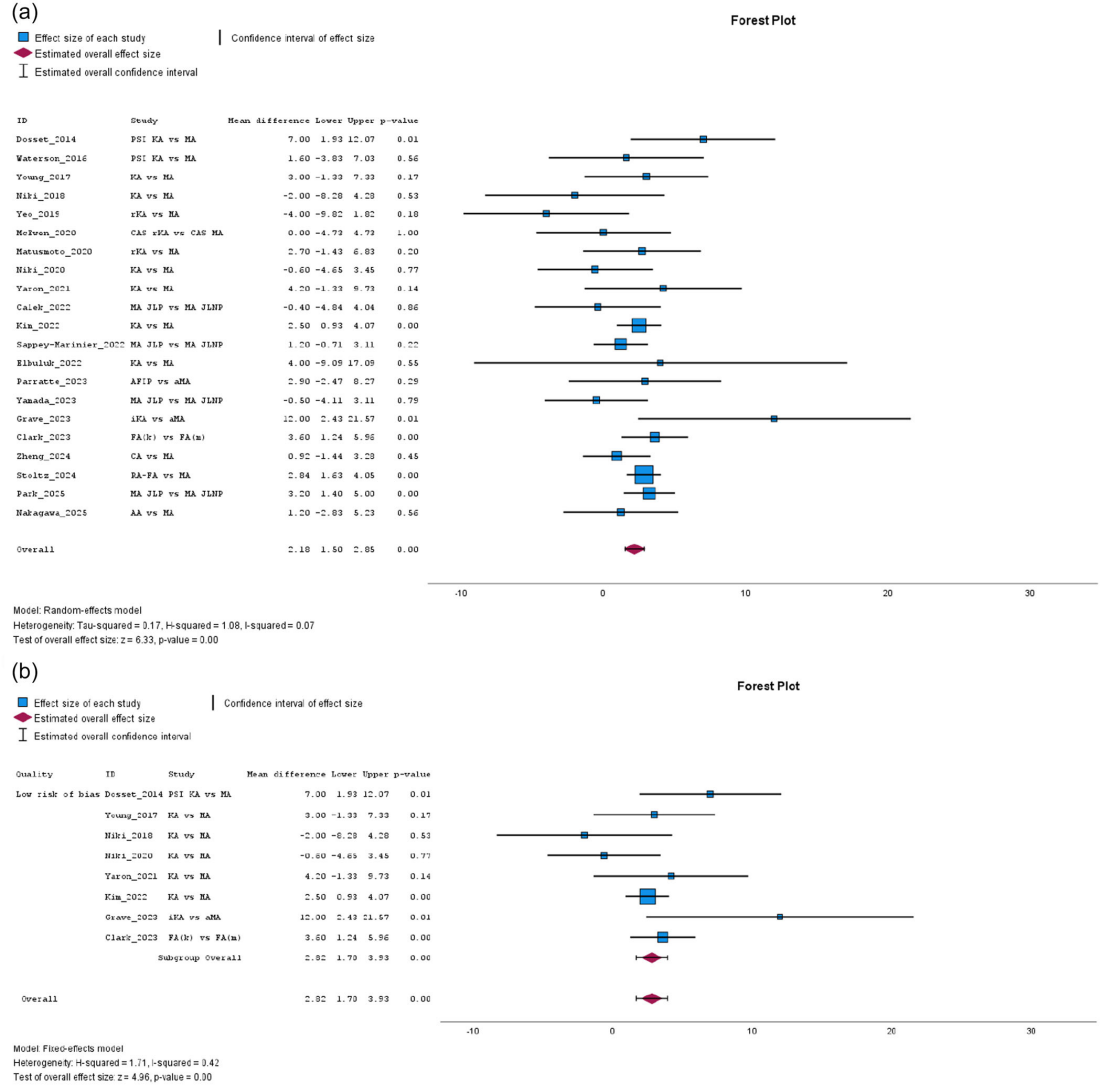
(a) Meta‐analysis forest plot for the range of motion (ROM). (b) Low risk of bias subgroup meta‐analysis for the ROM.

The ROM, from 21 studies [4, 8, 11, 16, 18, 32, 41, 42, 48, 52, 53, 57, 58, 65, 72, 77, 78, 79, 80, 81, 83], significantly favoured JLP, with low heterogeneity (MD: 2.18, 95% CI: 1.50–2.85, *p* = 0.00, *I*² = 7%, see Figure 8a). Subgroup analysis of studies with low risk of bias reinforced statistical significance in favour of JLP, with low heterogeneity (MD: 2.82, 95% CI: 1.70–3.93, *p* = 0.00, *I*² = 42%, see Figure 8b). Improvement in ROM, analysed in 17 studies [4, 8, 11, 16, 32, 41, 42, 48, 52, 53, 57, 58, 72, 79, 80, 81, 83], kept supporting JLP (MD: 2.12, 95% CI: 0.64–3.61, *p* = 0.01, *I*² = 44%), as well as the subgroup analysis of low risk studies (MD: 2.48, 95% CI: 0.73–4.24, *p* = 0.01, *I*² = 0%), with low and no heterogeneity, respectively.

Detailed forest plots for all outcomes are available in Supporting Information: File S1.

## DISCUSSION

Fair evidence supports preservation of joint line orientation in enhancing patient‐reported outcome measures in TKA. This systematic review and meta‐analysis provides a focused and up‐to‐date synthesis of the available evidence on this important factor. A relevant strength of this review is the inclusion of numerous recent studies, primarily from 2020 to 2025 [4, 8, 10, 11, 14, 18, 30, 31, 32, 37, 41, 42, 53, 58, 60, 65, 70, 71, 78, 79, 82], underscoring the growing interest and clinical relevance of joint line orientation in the pursuit of optimal TKA outcomes.

Across the quantitative analysis, JLP was consistently associated with statistically significant improvements in PROMs and functional measures. Notably, FJS, KOOS, KOOS JR, Knee Function – KSS 2011, OKS, and ROM all demonstrated significant benefits in favour of the JLP approach. A crucial trend observed was that subgroup analysis of studies with low and low‐to‐moderate risk of bias further amplified the significance of JLP benefits in several key measures, with greater improvements seen in FJS, Knee Function‐KSS, WOMAC, KOOS JR and ROM. These findings further emphasise the clinical relevance of joint line preservation, as lower‐risk studies employing more precise JLP techniques and robust methodological designs provide stronger, less biased evidence supporting JLP, thereby validating its clinical benefits.

The contrasting findings between the Function Scores of the KSS 1989 [29] and KSS 2011 [68] versions in our meta‐analysis provide valuable insight into the potential impact of preserving joint line orientation. While the KSS 1989 Function Score did not show a statistically significant advantage for JLP, the KSS 2011 version consistently demonstrated a significant benefit. This discrepancy may be explained by the fundamental differences between the two scoring systems. The 1989 version focuses on basic mobility metrics—such as walking distance, stair climbing, and use of walking aids—whereas the 2011 version encompasses a broader range of patient‐reported, real‐world activities, including kneeling, squatting, rising from a chair, and participation in recreational or athletic pursuits. As such, the KSS 2011 may be more sensitive to capturing the types of functional improvements associated with JLP. These benefits appear particularly evident in higher‐level activities that rely more heavily on joint mechanics and proprioception. This interpretation is further supported by several gait analysis studies included in our qualitative synthesis [5, 31, 51, 57, 77, 78, 80], which reported enhanced knee stability, improved quadriceps engagement, and more physiological movement and force patterns in patients undergoing JLP.

While the pooled differences identified in this meta‐analysis did not exceed commonly cited minimum clinically important difference (MCID) thresholds—such as 13.7 for FJS [13], 5 for OKS [12], and 9.2 for KOOS [2] —this must be interpreted with caution. MCIDs are typically derived from within‐patient changes (pre‐ vs. post‐operative changes) aiming to define the smallest change in a score that a patient would identify as meaningful. However, in a meta‐analysis, the effect size reflects a between‐group difference, which is conceptually distinct from the within‐subject changes used to calculate MCIDs [7, 45, 62]. Applying within‐patient thresholds to between‐group data may therefore be methodologically inappropriate and potentially misleading. Additionally, MCID values vary depending on the calculation method (e.g., anchor‐based, distribution‐based and ROC analysis), and many thresholds aim to distinguish satisfied from unsatisfied patients rather than detect more nuanced differences among high‐functioning individuals [21, 25, 38, 64]. Several PROMs also exhibit ceiling effects, reducing their sensitivity to subtle functional improvements in patients with already good outcomes [17, 54]. This may partly explain why certain studies—such as those by Parratte et al. [64], Nakagawa et al. [38], and McEwen et al. [36]—reported no significant differences in PROM scores, yet observed greater patient satisfaction or preference in the JLP group. These findings suggest that while pooled PROM differences may appear statistically modest, joint line preservation could still offer meaningful clinical benefits, especially in high‐functioning patients.

Although JLO preservation may not have a substantial effect reducing reported dissatisfaction rates following TKA, it may play an important role in optimising already favourable outcomes. This effect is particularly relevant within the framework of the “forgotten joint” concept, which represents the highest standard of success in joint replacement. In this context, among the available PROMs, the FJS stands out as the most suitable for capturing such subtle yet meaningful improvements, particularly in patients with higher satisfaction levels [1, 33, 73, 74]. Notably, in this meta‐analysis, the FJS demonstrated the largest pooled mean difference in favour of joint line preservation, further supporting its sensitivity to functional improvements in this context.

It is also reasonable to hypothesise that patients whose native joint line orientation deviates more significantly from neutral [27] —such as alignment “outliers” with marked varus or valgus deformities—may experience greater functional consequences when JLO is not maintained, compared to patients with near‐neutral alignment. However, the absence of standardised phenotypic classification across studies limited our ability to explore this interaction.

This meta‐analysis is limited by the high heterogeneity observed in several pooled outcomes, particularly for PROMs like FJS, KSS and WOMAC (*I*² > 75%), likely due to methodological differences, patient variability, and inconsistent reporting. Although subgroup analyses by study quality reduced heterogeneity in some cases, variability persisted. More stable findings were observed for FJS (subgroup), OKS, KOOS, and ROM.

Another limitation is the focus on a single phenotypic feature (JLO), without accounting for broader phenotypic variations (e.g., functional phenotypes, HKA alignment or laxity). Additional confounding factors—such as osteoarthritis severity, surgical indication, baseline function and psychological status—were also inconsistently reported, potentially influencing outcomes.

Future studies should aim to establish more standardised phenotypic groupings [26] to better identify which patients benefit most from joint line preservation. Stratified analyses based on broader phenotype classifications and more comprehensive reporting of patient‐specific variables will be essential for advancing individualised approaches in TKA.

## CONCLUSION

This systematic review and meta‐analysis found that joint line preservation in TKA is associated with statistically significant improvements in PROMs and functional outcomes, particularly in studies with low or low‐to‐moderate risk of bias. These results support the relevance of joint line orientation in optimising outcomes, though its isolated effect may be limited without considering broader phenotypic alignment strategies.

## AUTHOR CONTRIBUTIONS

**Dúnio Jácome‐Pacheco**: Writing of protocol; article screening and selection; data extraction and analysis; writing of manuscript. **Tiago Torres**: Reviewer of protocol; article screening and selection; data extraction. **Gonçalo Rodrigues**: Reviewer of protocol; reviewer and writing of the final manuscript. **Pedro Diniz**: Reviewer of protocol; supervision of methodology; reviewer and writing of the final manuscript. **Francisco Guerra Pinto**: Writing of protocol; referee in case of disagreement in article screening and selection; reviewer and writing of the final manuscript. **António Camacho**: Supervision of methodology and reviewing process; reviewer and writing of the final manuscript. **João Gamelas**: Supervision of methodology; reviewer and writing of the final manuscript. **Romain Seil**: Supervision of methodology; reviewer and writing of the final manuscript. **Michael Hirschmann**: Supervision of methodology; reviewer and writing of the final manuscript.

## CONFLICT OF INTEREST STATEMENT

The authors declare no conflicts of interest.

## ETHICS STATEMENT

PROSPERO registration number CRD42023473589.

## Supporting information

Supporting information.

## Data Availability

The data that support the findings of this study are available from the corresponding author upon reasonable request.

## References

[1] Adriani M , Malahias MA , Gu A , Kahlenberg CA , Ast MP , Sculco PK . Determining the validity, reliability, and utility of the forgotten joint score: a systematic review. J Arthroplasty. 2020;35:1137–1144. 31806559 10.1016/j.arth.2019.10.058

[2] Ayers DC , Yousef M , Yang W , Zheng H . Age‐related differences in pain, function, and quality of life following primary total knee arthroplasty: results from a FORCE‐TJR (Function and Outcomes Research for Comparative Effectiveness in Total Joint Replacement) Cohort. J Arthroplasty. 2023;38:S169–S176. 10.1016/j.arth.2023.04.005 37121490

[3] Bae K , Lee BS , Kim JM , Bin SI , Lee J , Kim D , et al. Effect of joint‐line obliquity on long‐term survivorship of total knee arthroplasty: a postoperative phenotype analysis. Knee Surg Sports Traumatol Arthrosc. 2024;32:3230–3238. 38895851 10.1002/ksa.12311

[4] Bar Ziv Y , Small I , Keidan T , Beit Ner E , Agar G , Shohat N . Patients undergoing staged bilateral knee arthroplasty are less aware of their kinematic aligned knee compared to their mechanical knee. J Orthop. 2021;23:155–159. 33542593 10.1016/j.jor.2020.12.032 PMC7840796

[5] Blakeney W , Clément J , Desmeules F , Hagemeister N , Rivière C , Vendittoli PA . Kinematic alignment in total knee arthroplasty better reproduces normal gait than mechanical alignment. Knee Surg Sports Traumatol Arthrosc. 2019;27:1410–1417. 30276435 10.1007/s00167-018-5174-1

[6] Borenstein M , Hedges LV , Higgins JPT , Rothstein HR . Introduction to Meta‐Analysis. Chichester, UK: Wiley; 2009.

[7] Boyer CW , Lee IE , Tenan MS . All MCIDs are wrong, but some may be useful. J Orthop Sports Phys Ther. 2022;52:401–407. 35647882 10.2519/jospt.2022.11193

[8] Calek AK , Ladurner A , Jud L , Zdravkovic V , Behrend H . Tibial joint line orientation has no effect on joint awareness after mechanically aligned total knee arthroplasty. Knee Surg Sports Traumatol Arthrosc. 2022;30:389–396. 34417835 10.1007/s00167-021-06696-4

[9] Calliess T , Bauer K , Stukenborg‐Colsman C , Windhagen H , Budde S , Ettinger M . PSI kinematic versus non‐PSI mechanical alignment in total knee arthroplasty: a prospective, randomized study. Knee Surg Sports Traumatol Arthrosc. 2017;25:1743–1748. 27120192 10.1007/s00167-016-4136-8

[10] Cherches M , Coss N , Nguyen K , Halvorson R , Allahabadi S , Bini S . No correlation between clinical outcomes and changes in the tibia‐metaphyseal angle following total knee arthroplasty: a retrospective study. J Arthroplasty. 2022;37:1793–1798. 35469985 10.1016/j.arth.2022.04.024

[11] Clark GW , Steer RA , Khan RN , Collopy DM , Wood D . Maintaining joint line obliquity optimizes outcomes of functional alignment in total knee arthroplasty in patients with constitutionally varus knees. J Arthroplasty. 2023;38:S239–S244. 37061140 10.1016/j.arth.2023.04.004

[12] Clement ND , MacDonald D , Simpson AHRW . The minimal clinically important difference in the Oxford knee score and Short Form 12 score after total knee arthroplasty. Knee Surg Sports Traumatol Arthrosc. 2014;22:1933–1939. 24253376 10.1007/s00167-013-2776-5

[13] Clement ND , Scott CEH , Hamilton DF , MacDonald D , Howie CR . Meaningful values in the Forgotten Joint Score after total knee arthroplasty. Bone Joint J. 2021;103–B:846–854. 10.1302/0301-620X.103B5.BJJ-2020-0396.R1 33934639

[14] D'Amato M , Kosse NM , Wymenga AB . Restoration of pre‐operative joint line orientation and alignment does not affect KSS and KOOS 1 year after total knee arthroplasty. Knee Surg Sports Traumatol Arthrosc. 2021;29:3170–3177. 32556430 10.1007/s00167-020-06097-z

[15] DeFrance MJ , Scuderi GR . Are 20% of patients actually dissatisfied following total knee arthroplasty? A systematic review of the literature. J Arthroplasty. 2023;38:594–599. 36252743 10.1016/j.arth.2022.10.011

[16] Dossett HG , Estrada NA , Swartz GJ , Lefevre GW , Kwasman BG , Dossett νHG , et al. A randomised controlled trial of kinematically and mechanically aligned total knee replacements: two‐year clinical results. Bone Joint J. 2014;1:96–907. 10.1302/0301-620X.96B7.32812 24986944

[17] Eckhard L , Munir S , Wood D , Talbot S , Brighton R , Walter B , et al. The ceiling effects of patient reported outcome measures for total knee arthroplasty. Orthop Traumatol: Surg Res. 2021;107:102758. 33316442 10.1016/j.otsr.2020.102758

[18] Elbuluk AM , Jerabek SA , Suhardi VJ , Sculco PK , Ast MP , Vigdorchik JM . Head‐to‐head comparison of kinematic alignment versus mechanical alignment for total knee arthroplasty. J Arthroplasty. 2022;37:S849–S851. 35093548 10.1016/j.arth.2022.01.052

[19] Ettinger M , Tuecking LR , Savov P , Windhagen H . Higher satisfaction and function scores in restricted kinematic alignment versus mechanical alignment with medial pivot design total knee arthroplasty: a prospective randomised controlled trial. Knee Surg Sports Traumatol Arthrosc. 2024;32:1275–1286. 38501253 10.1002/ksa.12143

[20] Franceschetti E , Campi S , Giurazza G , Tanzilli A , Gregori P , Laudisio A , et al. Mechanically aligned total knee arthroplasty does not yield uniform outcomes across all coronal plane alignment of the knee (CPAK) phenotypes. Knee Surg Sports Traumatol Arthrosc. 2024;32:3261–3271. 38984905 10.1002/ksa.12349

[21] Franceschini M , Boffa A , Pignotti E , Andriolo L , Zaffagnini S , Filardo G . The minimal clinically important difference changes greatly based on the different calculation methods. Am J Sports Med. 2023;51:1067–1073. 36811558 10.1177/03635465231152484 PMC10026158

[22] Gibbons JP , Zeng N , Bayan A , Walker ML , Farrington B , Young SW . No difference in 10‐year clinical or radiographic outcomes between kinematic and mechanical alignment in TKA: a randomized trial. Clin Orthop Related Res. 2025;483:140–149. 10.1097/CORR.0000000000003193 PMC11658733 39145997

[23] Higgins JPT . Cochrane Handbook for Systematic Reviews of Interventions, Version 6.5. 2024.

[24] Hirschmann MT , Becker R . The Unhappy Total Knee Replacement. Cham, Switzerland: Springer; 2015.

[25] Hirschmann MT , Bonnin MP . Abandon the mean value thinking: personalized medicine an intuitive way for improved outcomes in orthopaedics. Knee Surg Sports Traumatol Arthrosc. 2024;32:3129–3132. 39403804 10.1002/ksa.12503

[26] Hirschmann MT , Moser LB , Amsler F , Behrend H , Leclerq V , Hess S . Functional knee phenotypes: a novel classification for phenotyping the coronal lower limb alignment based on the native alignment in young non‐osteoarthritic patients. Knee Surg Sports Traumatol Arthrosc. 2019;27:1394–1402. 30976825 10.1007/s00167-019-05509-z

[27] Hirschmann MT , Khan ZA , Sava MP , von Eisenhart‐Rothe R , Graichen H , Vendittoli PA , et al. Definition of normal, neutral, deviant and aberrant coronal knee alignment for total knee arthroplasty. Knee Surg Sports Traumatol Arthrosc. 2024;32:473–489. 38293728 10.1002/ksa.12066

[28] Hörlesberger N , Zinggl C , Smolle MA , Leitner L , Lohberger B , Leithner A , et al. Mechanically aligned total knee arthroplasty with the extension‐first technique does not equally restore neutral knee alignment in all preoperative knee phenotypes. Knee Surg Sports Traumatol Arthrosc. 2023;31:1405–1411. 36087129 10.1007/s00167-022-07147-4 PMC10049937

[29] Insall JN , Dorr LD , Scott RD , Norman W . Rationale of the Knee Society clinical rating system. Clin Orthop Relat Res. 1989;248:13–14. 2805470

[30] Jeremić DV , Massouh WM , Sivaloganathan S , Rosali AR , Haaker RG , Rivière C . Short‐term follow‐up of kinematically vs. mechanically aligned total knee arthroplasty with medial pivot components: a case‐control study. Orthop Traumatol: Surg Res. 2020;106:921–927. 32522532 10.1016/j.otsr.2020.04.005

[31] Kaneda K , Niki Y , Kuroyanagi Y , Kobayashi S , Harato K , Iwama Y , et al. Kinematically aligned total knee arthroplasty using medial pivot knee prosthesis enhances medial pivot motion: a comparative kinematic study with mechanically aligned total knee arthroplasty. Arthroplast Today. 2022;13:24–28. 34917717 10.1016/j.artd.2021.10.004 PMC8666599

[32] Kim TW , Lee JI , Choi HG , Yoo HJ , Kim KT , Lee YS . Comparison of the radiologic, morphometric, and clinical outcomes between kinematically and mechanically aligned total knee arthroplasty: a propensity matching study. J Knee Surg. 2022;35:1453–1461. 33657622 10.1055/s-0041-1725006

[33] Kuhns BD , Harris WT , Domb BG . Low ceiling effects of the Forgotten Joint Score compared with legacy measures after joint‐preserving procedures: a systematic review. Arthroscopy. 2023;39:2086–2095. 36804458 10.1016/j.arthro.2023.01.107

[34] Laende EK , Richardson CG , Dunbar MJ . A randomized controlled trial of tibial component migration with kinematic alignment using patient‐specific instrumentation versus mechanical alignment using computer‐assisted surgery in total knee arthroplasty. Bone Joint J. 2019;101:929–940. 31362561 10.1302/0301-620X.101B8.BJJ-2018-0755.R3

[35] Lange JK , Lee YY , Spiro SK , Haas SB . Satisfaction rates and quality of life changes following total knee arthroplasty in age‐differentiated cohorts. J Arthroplasty. 2018;33:1373–1378. 29395722 10.1016/j.arth.2017.12.031

[36] Lee JH , Kwon SC , Hwang JH , Lee JK , Kim JI . Functional alignment maximises advantages of robotic arm‐assisted total knee arthroplasty with better patient‐reported outcomes compared to mechanical alignment. Knee Surg Sports Traumatol Arthrosc. 2024;32:896–906. 38454836 10.1002/ksa.12120

[37] MacDessi SJ , Griffiths‐Jones W , Chen DB , Griffiths‐Jones S , Wood JA , Diwan AD , et al. Restoring the constitutional alignment with a restrictive kinematic protocol improves quantitative soft‐tissue balance in total knee arthroplasty: a randomized controlled trial. Bone Joint J. 2020;102:117–124. 31888372 10.1302/0301-620X.102B1.BJJ-2019-0674.R2 PMC6974544

[38] Maltenfort M , Díaz‐Ledezma C . Statistics in brief: minimum clinically important difference‐availability of reliable estimates. Clin Orthop Relat Res. 2017;475:933–946. 28050812 10.1007/s11999-016-5204-6 PMC5339150

[39] Mathis DT , Hauser A , Iordache E , Amsler F , Hirschmann MT . Typical pain patterns in unhappy patients after total knee arthroplasty. J Arthroplasty. 2021;36:1947–1957. 33583666 10.1016/j.arth.2021.01.040

[40] Matsumoto T , Takayama K , Ishida K , Hayashi S , Hashimoto S , Kuroda R . Radiological and clinical comparison of kinematically versus mechanically aligned total knee arthroplasty. Bone Joint J. 2017;99–B(5):640–646. 10.1302/0301-620X.99B5.BJJ-2016-0688.R2 28455473

[41] Matsumoto T , Takayama K , Ishida K , Kuroda Y , Tsubosaka M , Muratsu H , et al. Intraoperative soft tissue balance/kinematics and clinical evaluation of modified kinematically versus mechanically aligned total knee arthroplasty. J Knee Surg. 2020;33:777–784. 31067590 10.1055/s-0039-1688504

[42] McEwen PJ , Dlaska CE , Jovanovic IA , Doma K , Brandon BJ . Computer‐assisted kinematic and mechanical axis total knee arthroplasty: a prospective randomized controlled trial of bilateral simultaneous surgery. J Arthroplasty. 2020;35:443–450. 31591010 10.1016/j.arth.2019.08.064

[43] McGrath S , Zhao X , Steele R , Thombs BD , Benedetti A , Levis B , et al. Estimating the sample mean and standard deviation from commonly reported quantiles in meta‐analysis. Stat Methods Med Res. 2020;29:2520–2537. 32292115 10.1177/0962280219889080 PMC7390706

[44] Moher D , Liberati A , Tetzlaff J , Altman DG . Preferred reporting items for systematic reviews and meta‐analyses: the PRISMA statement. PLoS Med. 2009;6:e1000097. 19621072 10.1371/journal.pmed.1000097 PMC2707599

[45] Molino J , Harrington J , Racine‐Avila J , Aaron R . Deconstructing the minimum clinically important difference (MCID). Orthop Res Rev. 2022;14:35–42. 35210873 10.2147/ORR.S349268 PMC8860454

[46] Muertizha M , Cai X , Ji B , Aimaiti A , Cao L . Factors contributing to 1‐year dissatisfaction after total knee arthroplasty: a nomogram prediction model. J Orthop Surg. 2022;17:367. 10.1186/s13018-022-03205-2 PMC9330701 35902950

[47] Murakami K , Hamai S , Okazaki K , Ikebe S , Higaki H , Shimoto T , et al. Preoperative tibial mechanical axis orientation and articular surface design influence on the coronal joint line orientation relative to the ground during gait after total knee arthroplasties. Knee Surg Sports Traumatol Arthrosc. 2018;26:3368–3376. 29556891 10.1007/s00167-018-4899-1

[48] Nakagawa Y , Koga H , Sekiya I , Hasegawa S , Katagiri H , Watanabe T . Equivalent clinical outcomes between anatomical alignment versus mechanical alignment of simultaneous bilateral total knee arthroplasty using a posterior‐stabilized prosthesis during an average follow‐up of five years: a prospective randomized clinical trial. J Arthroplasty. 2025;40:84–91. 39025273 10.1016/j.arth.2024.07.014

[49] Nakajima A , Sonobe M , Akatsu Y , Aoki Y , Takahashi H , Suguro T , et al. Association between limb alignment and patient‐reported outcomes after total knee arthroplasty using an implant that reproduces anatomical geometry. J Orthop Surg. 2018;13:320. 10.1186/s13018-018-1030-8 PMC6296124 30558616

[50] Nakano N , Shoman H , Olavarria F , Matsumoto T , Kuroda R , Khanduja V . Why are patients dissatisfied following a total knee replacement? A systematic review. Int Orthop. 2020;44:1971–2007. 32642827 10.1007/s00264-020-04607-9 PMC7584563

[51] Niki Y , Nagura T , Nagai K , Kobayashi S , Harato K . Kinematically aligned total knee arthroplasty reduces knee adduction moment more than mechanically aligned total knee arthroplasty. Knee Surg Sports Traumatol Arthrosc. 2017;26:1629–1635. 29204861 10.1007/s00167-017-4788-z

[52] Niki Y , Kobayashi S , Nagura T , Udagawa K , Harato K . Joint line modification in kinematically aligned total knee arthroplasty improves functional activity but not patient satisfaction. J Arthroplasty. 2018;33:2125–2130. 29506930 10.1016/j.arth.2018.02.015

[53] Niki Y , Nagura T , Kobayashi S , Udagawa K , Harato K . Who will benefit from kinematically aligned total knee arthroplasty? perspectives on patient‐reported outcome measures. J Arthroplasty. 2020;35:438–442.e2. 31668528 10.1016/j.arth.2019.09.035

[54] Ostojić M , Violante B , Becker R , Hirschmann MT , Indelli PF . Patient‐reported outcome measures, the holy grail of outcome assessment: are they powerful enough to show a difference in knee arthroplasty alignment? A call for more comprehensive and objective data collection. Knee Surg Sports Traumatol Arthrosc. 2025;33:397–400. 39550621 10.1002/ksa.12510

[55] Ouzzani M , Hammady H , Fedorowicz Z , Elmagarmid A . Rayyan‐a web and mobile app for systematic reviews. Syst Rev. 2016;5:210. 27919275 10.1186/s13643-016-0384-4 PMC5139140

[56] Pangaud C , Siboni R , Gonzalez JF , Argenson JN , Seil R , Froidefond P , et al. Restoring the preoperative phenotype according to the coronal plane alignment of the knee classification after total knee arthroplasty leads to better functional results. J Arthroplasty. 2024;39:2970–2976. 38880407 10.1016/j.arth.2024.06.012

[57] Park SY , Cho JH , Nam HS , Ho JPY , Lee YS . Bi‐cruciate stabilized total knee arthroplasty restores the native knee alignments better than conventional posterior stabilized total knee arthroplasty. Arch Orthop Trauma Surg. 2024;145:31. 39666122 10.1007/s00402-024-05714-7

[58] Parratte S , Van Overschelde P , Bandi M , Ozturk BY , Batailler C . An anatomo‐functional implant positioning technique with robotic assistance for primary TKA allows the restoration of the native knee alignment and a natural functional ligament pattern, with a faster recovery at 6 months compared to an adjusted mechanical technique. Knee Surg Sports Traumatol Arthrosc. 2023;31:1334–1346. 35552475 10.1007/s00167-022-06995-4

[59] Pratobevera A , Seil R , Menetrey J . Joint line and knee osteotomy. EFORT Open Rev. 2024;9:375–386. 38726996 10.1530/EOR-24-0037 PMC11099584

[60] Rak D , Klann L , Heinz T , Anderson P , Stratos I , Nedopil AJ , et al. Influence of mechanical alignment on functional knee phenotypes and clinical outcomes in primary TKA: a 1‐year prospective analysis. J Pers Med. 2023;13:778. 37240948 10.3390/jpm13050778 PMC10222901

[61] Rames RD , Mathison M , Meyer Z , Barrack RL , Nam D . No impact of under‐correction and joint line obliquity on clinical outcomes of total knee arthroplasty for the varus knee. Knee Surg Sports Traumatol Arthrosc. 2018;26:1506–1514. 28299385 10.1007/s00167-017-4507-9

[62] Riddle DL , Dumenci L . Limitations of minimal clinically important difference estimates and potential alternatives. J Bone Jt Surg. 2024;106:931–937. 10.2106/JBJS.23.00467 38060688

[63] Rodríguez AO , Jagota I , Baré J , Shimmin A . Impact of changes in native coronal plane alignment of the knee (CPAK) on patient‐reported outcome measures (PROMS). A bilateral single implant study. J Orthop. 2025;65:64–70. 39801902 10.1016/j.jor.2024.12.002 PMC11721536

[64] Rossi MJ , Brand JC , Lubowitz JH . Minimally clinically important difference (MCID) is a low bar. Arthroscopy. 2023;39:139–141. 36603986 10.1016/j.arthro.2022.11.001

[65] Sappey‐Marinier E , Batailler C , Swan J , Schmidt A , Cheze L , MacDessi SJ , et al. Mechanical alignment for primary TKA may change both knee phenotype and joint line obliquity without influencing clinical outcomes: a study comparing restored and unrestored joint line obliquity. Knee Surg Sports Traumatol Arthrosc. 2022;30:2806–2814. 34291311 10.1007/s00167-021-06674-w

[66] Schelker BL , Moret CS , Sava MP , von Eisenhart‐Rothe R , Graichen H , Arnold MP , et al. The impact of different alignment strategies on bone cuts in total knee arthroplasty for varus knee phenotypes. Knee Surg Sports Traumatol Arthrosc. 2023;31:1840–1850. 36811657 10.1007/s00167-023-07351-w PMC10089997

[67] Schelker BL , Moret CS , von Eisenhart‐Rothe R , Graichen H , Arnold MP , Leclercq V , et al. The impact of different alignment strategies on bone cuts for neutral knee phenotypes in total knee arthroplasty. Knee Surg Sports Traumatol Arthrosc. 2023;31:1267–1275. 36326877 10.1007/s00167-022-07209-7 PMC10050061

[68] Scuderi GR , Bourne RB , Noble PC , Benjamin JB , Lonner JH , Scott WN . The new Knee Society Knee Scoring system. Clin Orthop Relat Res. 2012;470:3–19. 22045067 10.1007/s11999-011-2135-0 PMC3237971

[69] Seil R , Pape D . Causes of failure and etiology of painful primary total knee arthroplasty. Knee Surg Sports Traumatol Arthrosc. 2011;19:1418–1432. 21833512 10.1007/s00167-011-1631-9

[70] Shelton TJ , Gill M , Athwal G , Howell SM , Hull ML . Outcomes in patients with a calipered kinematically aligned TKA that already had a contralateral mechanically aligned TKA. J Knee Surg. 2021;34:087–093. 10.1055/s-0039-1693000 31288274

[71] Shin KH , Jang KM , Han SB . Residual varus alignment can reduce joint awareness, restore joint parallelism, and preserve the soft tissue envelope during total knee arthroplasty for varus osteoarthritis. Knee Surg Sports Traumatol Arthrosc. 2022;30:507–516. 32743784 10.1007/s00167-020-06201-3

[72] Stoltz MJ , Smith NS , Abhari S , Whitaker J , Baker JF , Smith LS , et al. Patient‐reported outcomes in robotic‐assisted vs manual cementless total knee arthroplasty. Arthroplast Today. 2024;30:101488. 39822912 10.1016/j.artd.2024.101488 PMC11735922

[73] Thompson SM , Salmon LJ , Webb JM , Pinczewski LA , Roe JP . Construct validity and test re‐test reliability of the Forgotten Joint Score. J Arthroplasty. 2015;30:1902–1905. 26027525 10.1016/j.arth.2015.05.001

[74] Thomsen MG , Latifi R , Kallemose T , Barfod KW , Husted H , Troelsen A . Good validity and reliability of the forgotten joint score in evaluating the outcome of total knee arthroplasty. Acta Orthop. 2016;87:280–285. 26937689 10.3109/17453674.2016.1156934 PMC4900097

[75] Toyono S , Suzuki A , Nakajima T , Wanezaki Y , Aso M , Yamamoto T , et al. Knee joint line orientation after total knee arthroplasty is affected by the mechanical axis inclination of the lower limb according to foot position. J Joint Surg Res. 2023;1:123–127.

[76] Wan X , Wang W , Liu J , Tong T . Estimating the sample mean and standard deviation from the sample size, median, range and/or interquartile range. BMC Med Res Methodol. 2014;14:135. 25524443 10.1186/1471-2288-14-135 PMC4383202

[77] Waterson HB , Clement ND , Eyres KS , Mandalia VI , Toms AD . The early outcome of kinematic versus mechanical alignment in total knee arthroplasty: a prospective randomised control trial. Bone Joint J. 2016;98:B10. 10.1302/0301-620X.98B10.36862 27694590

[78] Winnock de Grave P , Van Criekinge T , Luyckx T , Moreels R , Gunst P , Claeys K . Restoration of the native tibial joint line obliquity in total knee arthroplasty with inverse kinematic alignment does not increase knee adduction moments. Knee Surg Sports Traumatol Arthrosc. 2023;31:4692–4704. 37311955 10.1007/s00167-023-07464-2

[79] Yamada M , Nakajima A , Sonobe M , Akatsu Y , Yamamoto K , Saito J , et al. The impact of postoperative inclination of the joint line on clinical outcomes in total knee arthroplasty using a prosthesis with anatomical geometry. Sci Rep. 2023;13:979. 36653469 10.1038/s41598-023-28182-2 PMC9849260

[80] Yeo JH , Seon JK , Lee DH , Song EK . No difference in outcomes and gait analysis between mechanical and kinematic knee alignment methods using robotic total knee arthroplasty. Knee Surg Sports Traumatol Arthrosc. 2019;27:1142–1147. 30220048 10.1007/s00167-018-5133-x

[81] Young SW , Walker ML , Bayan A , Briant‐Evans T , Pavlou P , Farrington B . The Chitranjan S. Ranawat Award: no difference in 2‐year functional outcomes using kinematic versus mechanical alignment in TKA: a randomized controlled clinical trial. Clin Orthop Relat Res. 2017;475:9–20. 27113595 10.1007/s11999-016-4844-x PMC5174030

[82] Young SW , Sullivan NPT , Walker ML , Holland S , Bayan A , Farrington B . No difference in 5‐year clinical or radiographic outcomes between kinematic and mechanical alignment in TKA: a randomized controlled trial. Clin Orthop Relat Res. 2020;478:1271–1279. 32039955 10.1097/CORR.0000000000001150 PMC7319387

[83] Zheng K , Wang Y , Wang T , Zhu F , Zhang L , Li R , et al. Restoration of constitutional alignment optimizes outcomes of computer navigated total knee arthroplasty: a prospective randomized controlled trial. Int Orthop. 2024;48:971–981. 38289379 10.1007/s00264-024-06093-9

