# Supplementary material for: Preserving joint line orientation in TKA improves short‐ to mid‐term outcomes: A systematic review and meta‐analysis
Source: J Exp Orthop. 2025 Oct 31;12(4):e70458. doi: 10.1002/jeo2.70458 (PMC12577764; doi:10.1002/jeo2.70458)

Supplementary File

Preserving Joint Line Orientation in TKA Improves Short- to Mid-Term Outcomes: A Systematic Review and Meta-Analysis

JEO (Journal of Experimental Orthopaedics)

Authors

Dúnio Jácome-Pacheco[1,2], Tiago Torres[1], Gonçalo Rodrigues[1], Pedro Diniz[3,4], Francisco Guerra-Pinto[1], António Camacho[2,5], João Gamelas[2,6], Romain Seil[3], Michael Hirschmann[7]

Affiliations

1. Hospital Ortopédico de Sant'Ana, Parede, Lisbon, Portugal.

2. NOVA Medical School, Universidade NOVA de Lisboa, Lisbon, Portugal.

3. Luxembourg Institute of Research in Orthopaedics, Sports Medicine and Science (LIROMS), Luxembourg, Luxembourg.

4. Department of Bioengineering, iBB – Institute for Bioengineering and Biosciences, Instituto Superior Técnico, Universidade de Lisboa, Lisbon, Portugal

5. Centro de Responsabilidade Integrado de Traumatologia Ortopédica (CRI-TO) do Centro Hospitalar Universitário de Lisboa Central (CHULC), Lisbon, Portugal.

6. Direção Clínica Hospitalar da Unidade Local de Saúde de Lisboa Ocidental, Lisbon, Portugal.

7. Department of Orthopaedic Surgery and Traumatology, Kantonsspital Baselland, Bruderholz, Switzerland

---

Corresponding Author

Name: Dúnio Jácome-Pacheco
Email: duniopacheco@gmail.com
Address: Hospital Ortopédico de Sant'Ana, Rua de Benguela, 501, 2775-028 Parede, Portugal

Estimates of mean and standard deviation were derived from reported medians, ranges, and/or interquartile ranges using the online tool developed by McGrath et al. (https://smcgrath.shinyapps.io/estmeansd/), which implements methods described in their 2020 publication [43].

Whenever only the mean and range were reported, the standard deviation (SD) was estimated using the method proposed by Wan [76] formula:

Formula 1

$$SD = \frac{b - a}{\Phi^{-1}\left(\frac{n-0.375}{n+0.25}\right) - \Phi^{-1}\left(\frac{0.375}{n+0.25}\right)}$$

SD – estimated standard deviation;
a – minimum value;
b – maximum value;
n – sample size;
Φ^{-1} – inverse cumulative distribution function (quantile function) of the standard normal distribution.

Standard Deviation (SD) was estimated from the Mean and P-value using Cochrane [23] formulas:

Formula 2

$$\mathrm{SD} = \frac{\mathrm{SE}}{\sqrt{\left(\frac{1}{N_E} + \frac{1}{N_C}\right)}}$$

Formula 3

$$SE = \frac{\mathrm{Mean}_A - \mathrm{Mean}_B}{t}$$

Formula 4

$$t = \mathrm{T.INV}(1 - \frac{p}{2}, df)$$

Formula 5

$$df = n_A + n_B - 2$$

df – degrees of freedom;
N_E – sample size in the joint line-preserving group;
N_C – sample size in the non-joint line preserving;
p – p-value;
SD – standard deviation;
SE – standard error;
t – t- value.

Mean and SD score improvement (I), if not provided, was calculated using the following formulas:

Formula 6

$$\mathrm{Improvement} = M_{\mathrm{post}} - M_{\mathrm{pre}}$$

Mpost = postoperative mean score;

Mpre = preoperative mean score;

Formula 7

$$SD_{\text{improvement}} = \sqrt{SD_{\text{pre}}^2 + SD_{\text{post}}^2 - 2c(SD_{\text{pre}})(SD_{\text{post}})}$$

SDpre = preoperative standard deviation;
SDpost = postoperative standard deviation;
c = correlation between preoperative and postoperative scores (0.5, if not provided).

Fig. 1.a Meta-analysis forest plot for the FJS

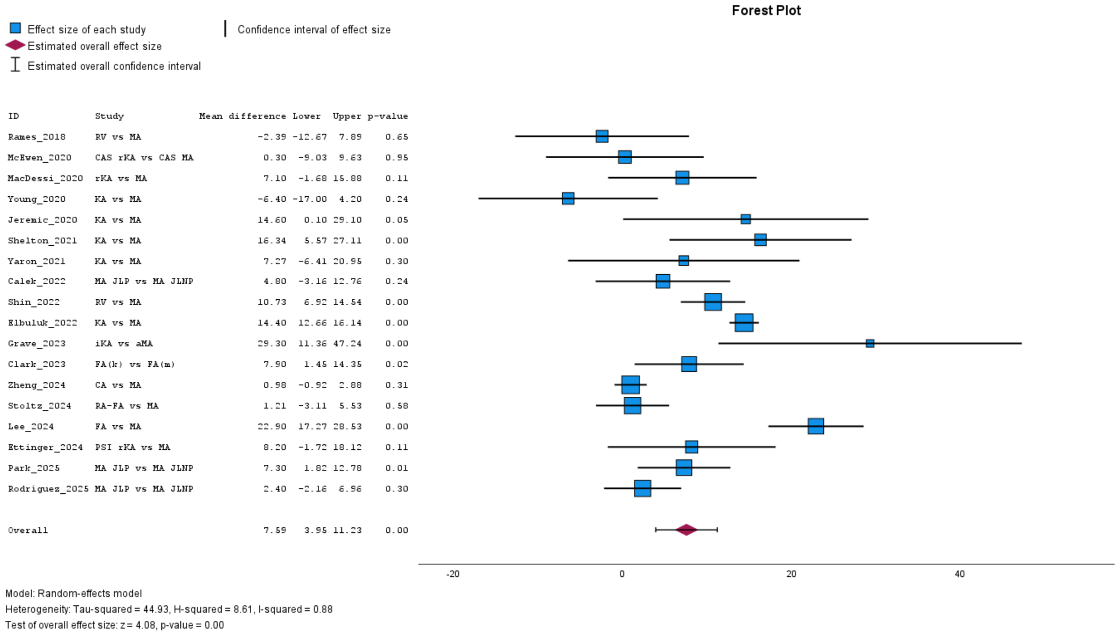

Fig. 1.b Low risk of bias subgroup meta-analysis for the FJS

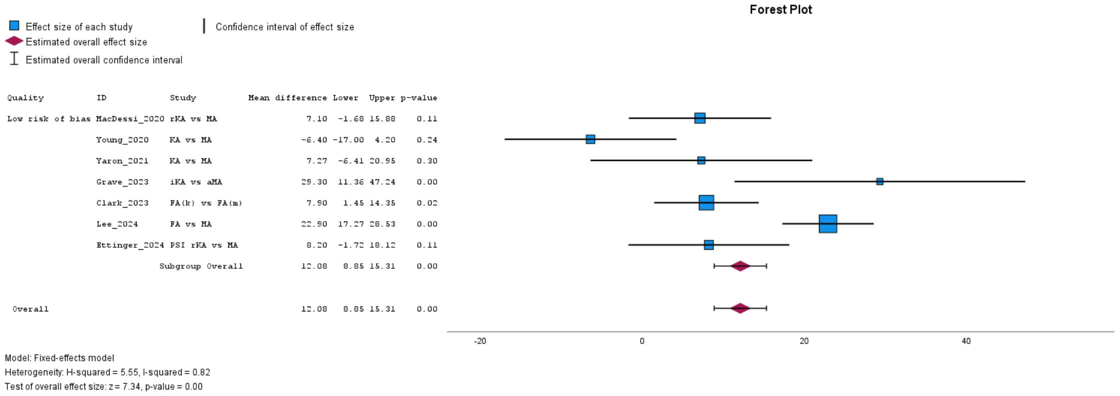

Fig. 1.c Meta-analysis forest plot for the FJS improvement

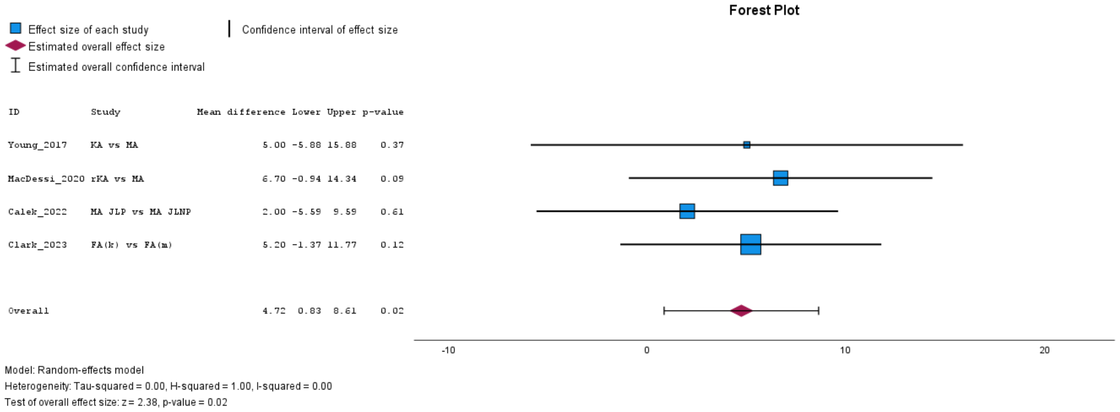

Fig. 1.d Low-to-moderate risk of bias subgroup meta-analysis for the FJS improvement

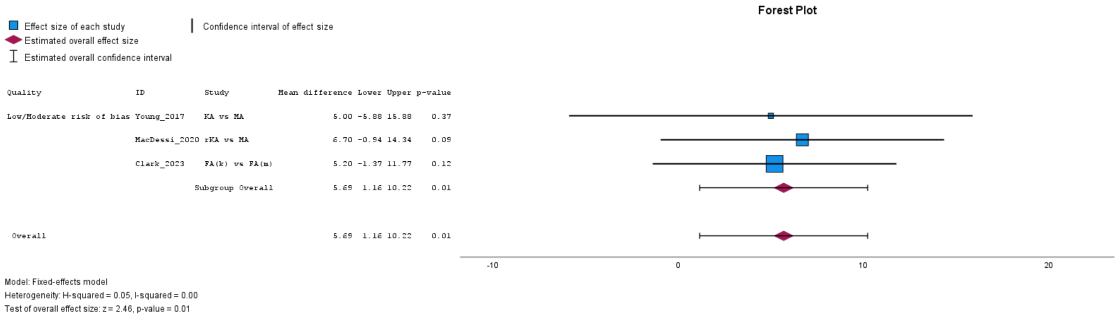

Fig. 2.a Forest plot assessing homogeneity between Knee Objective Indicators from KSS 1989 and KSS 2011 versions

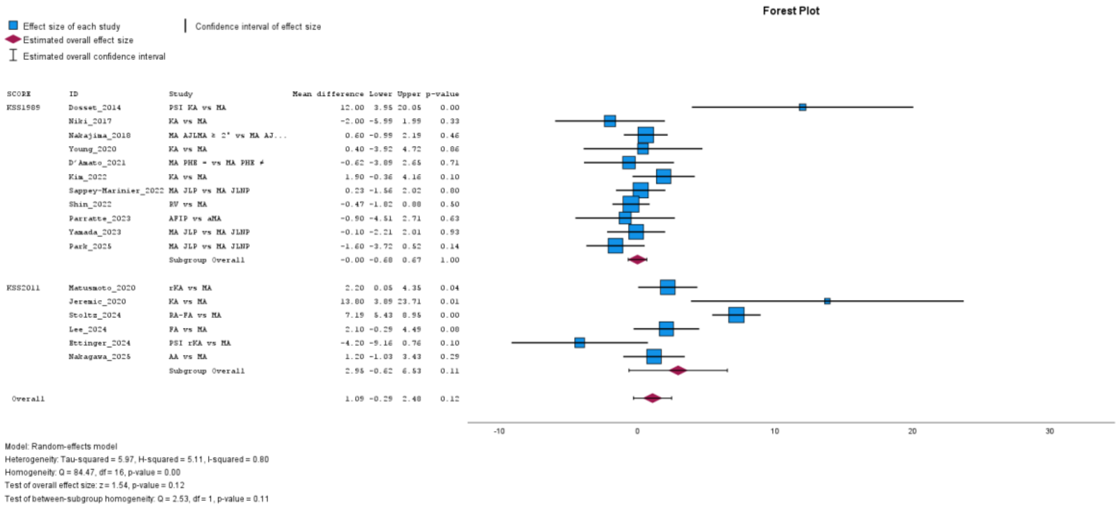

Fig. 2.b Meta-analysis forest plot for the Knee Objective Indicators-KSS

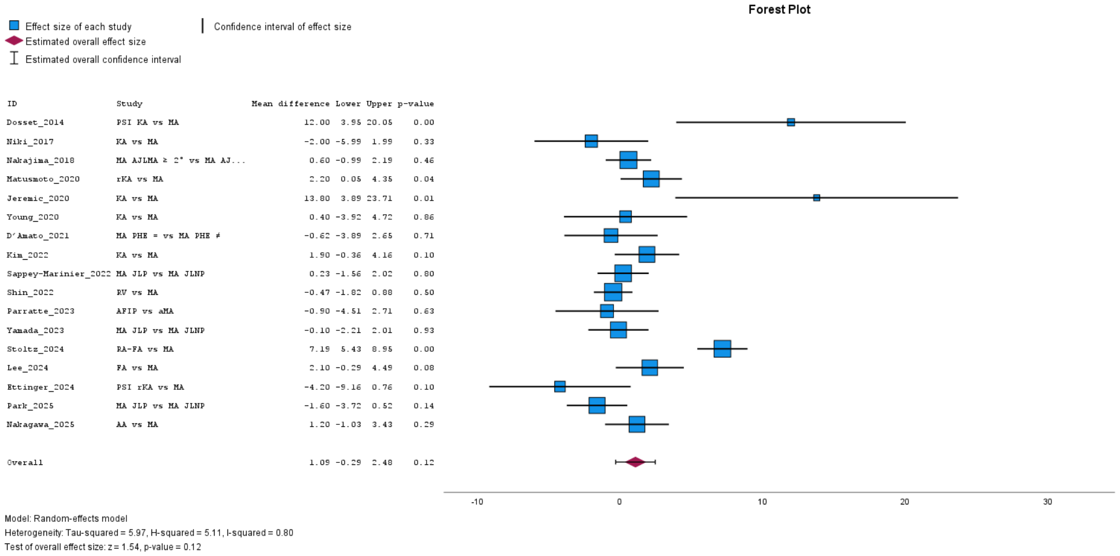

Fig. 2.c Low-to-moderate risk of bias subgroup meta-analysis for the Knee Objective Indicators-KSS

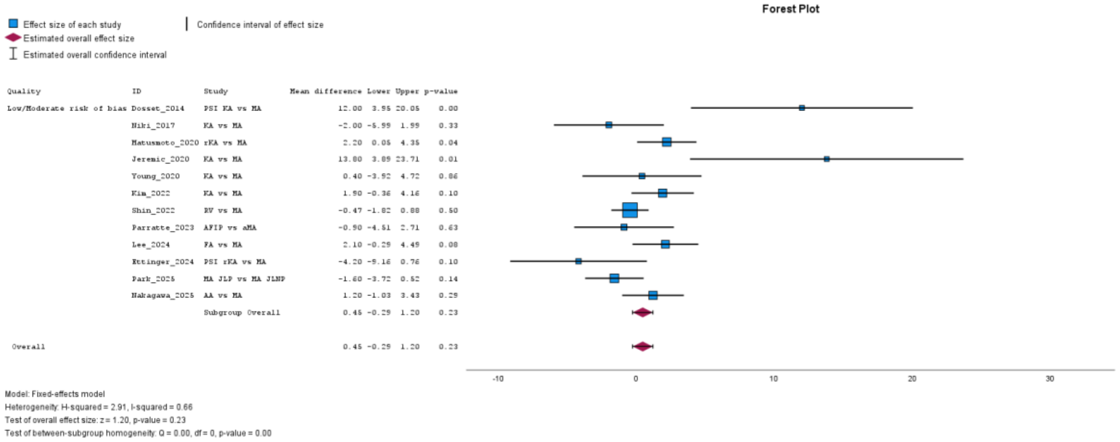

Fig. 2.d Meta-analysis forest plot for the Knee Objective Indicators-KSS improvement

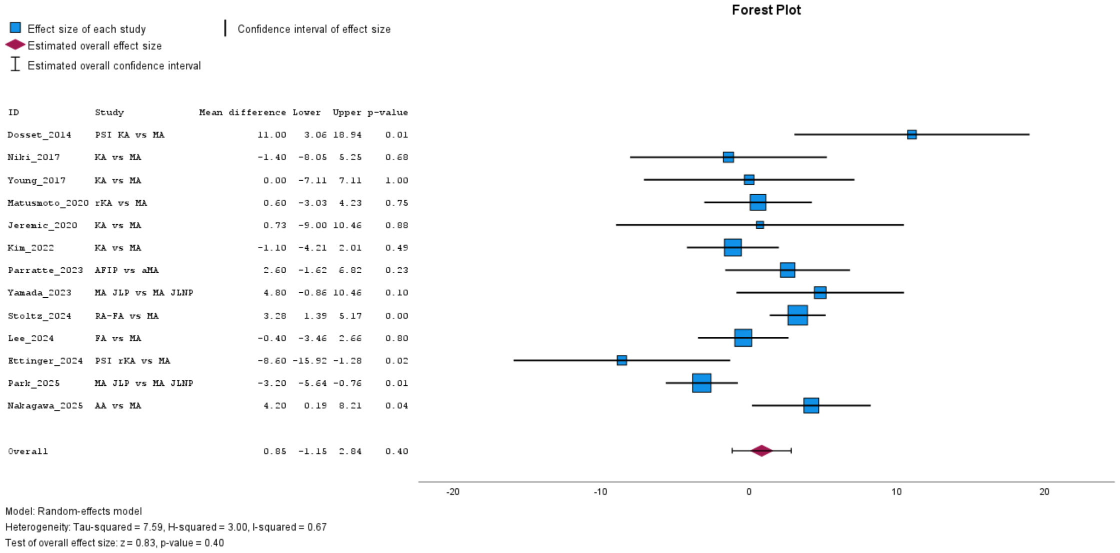

Fig. 2.e Low-to-moderate risk of bias subgroup meta-analysis for the Knee Objective Indicators-KSS improvement

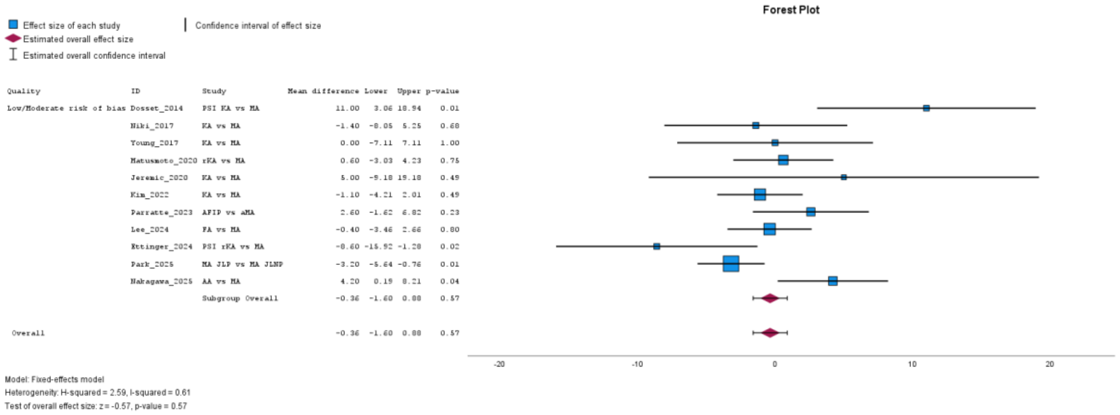

Fig. 3.a Forest plot assessing homogeneity between Function Score from KSS 1989 and KSS 2011 versions

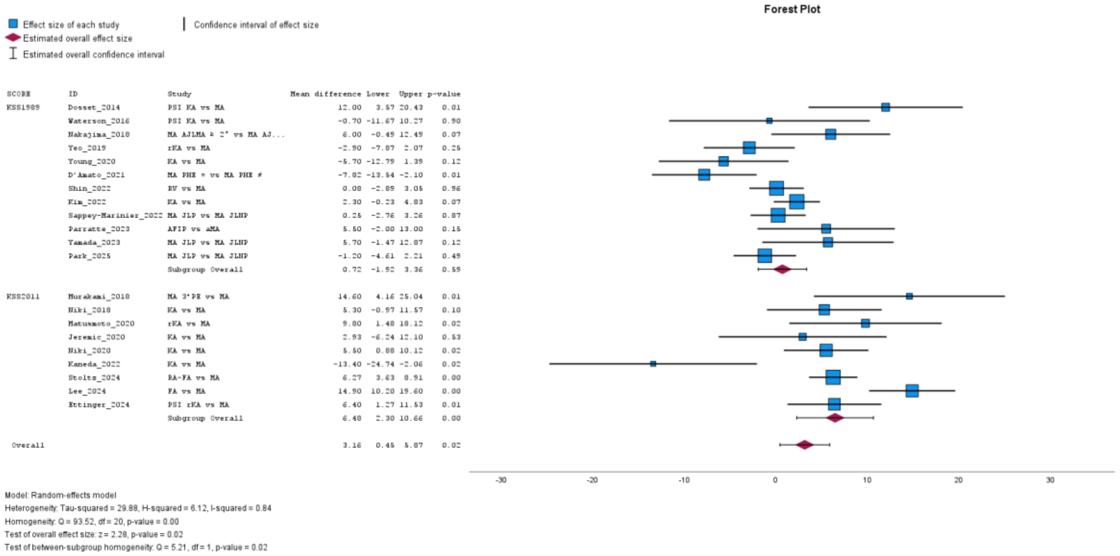

Fig. 3.b Meta-analysis forest plot for the Knee Function-KSS 1989

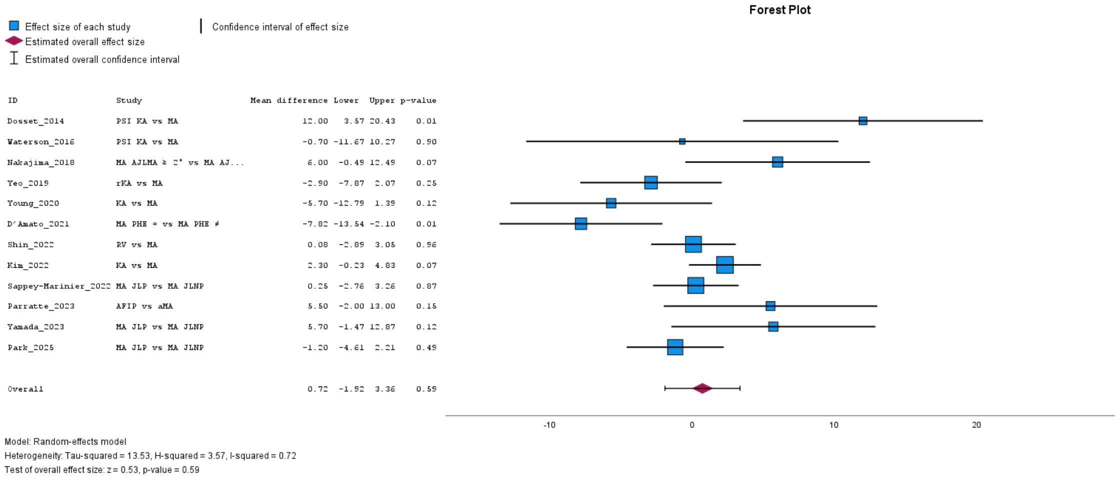

Fig. 3.c Low-to-moderate risk of bias subgroup meta-analysis for the Knee Function-KSS 1989

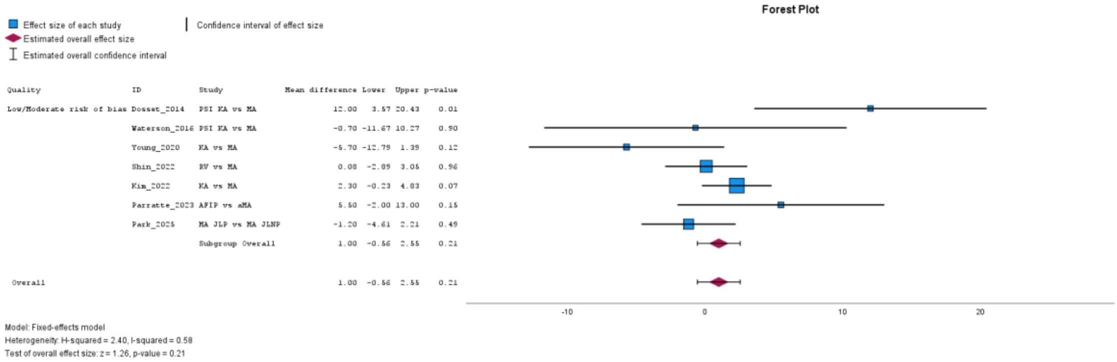

Fig. 3.d Meta-analysis forest plot for the Knee Function-KSS 2011

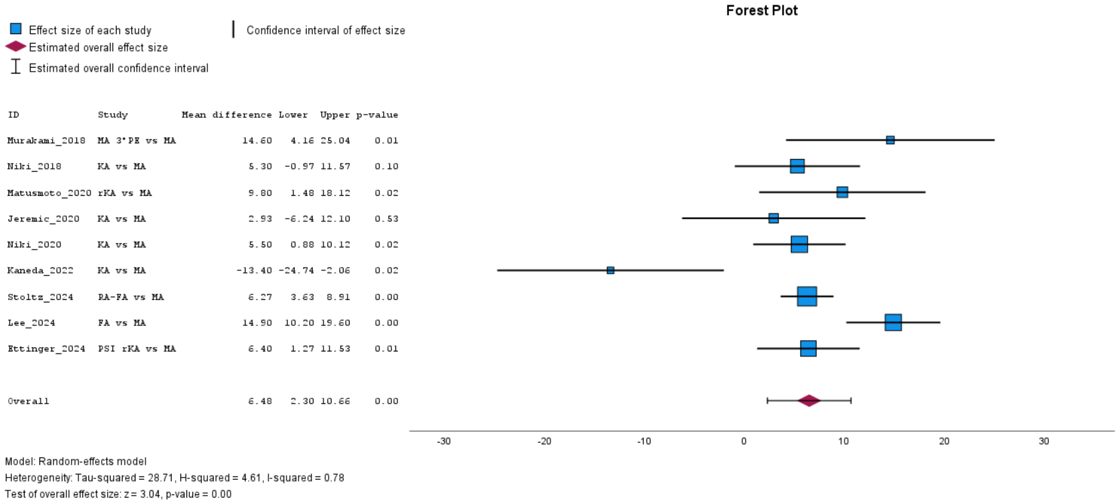

Fig. 3.e Low-to-moderate risk of bias subgroup meta-analysis for the Knee Function-KSS 2011

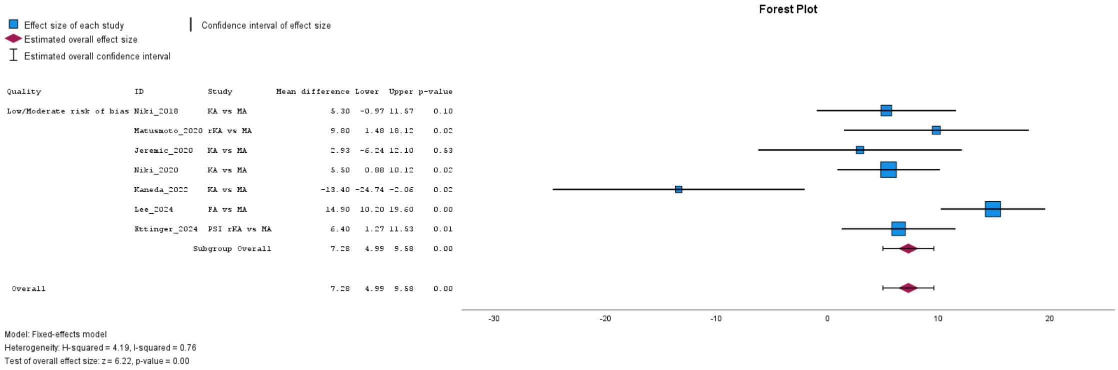

Fig. 3.f Meta-analysis forest plot for the knee function-KSS 1989 improvement

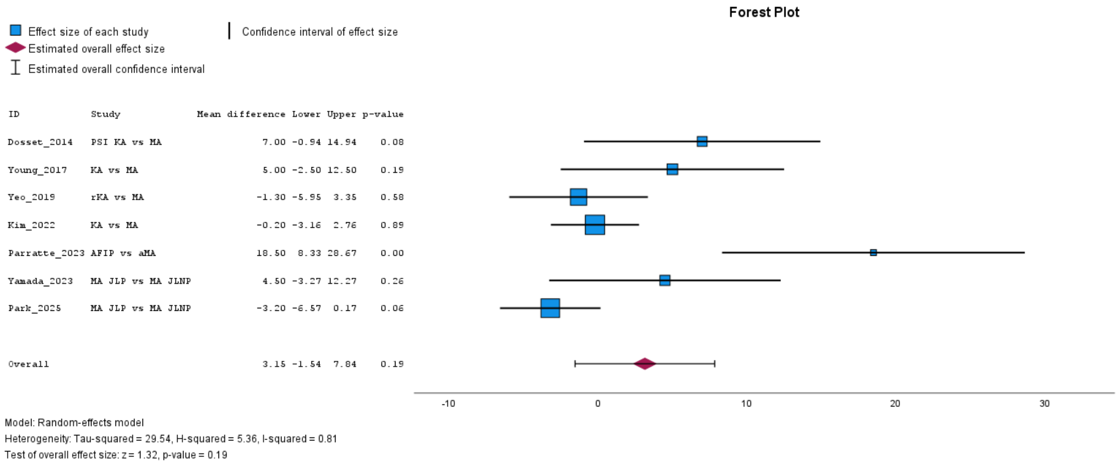

Fig. 3.g Low-to-moderate risk of bias subgroup meta-analysis for the knee function-KSS 1989 improvement

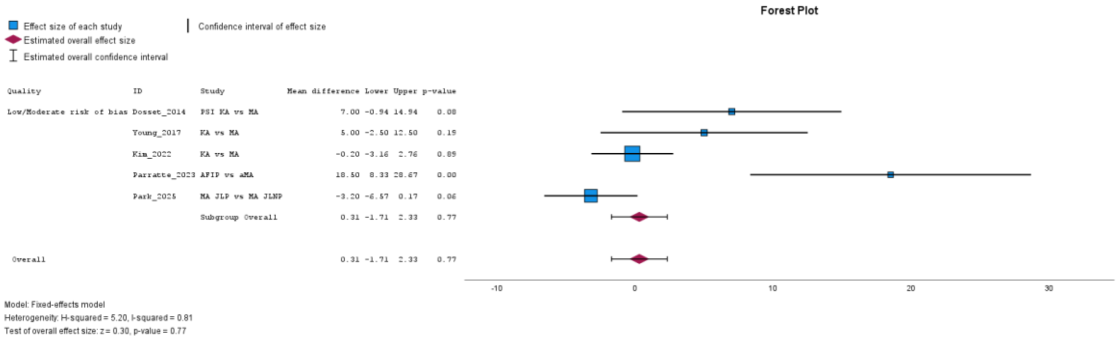

Fig. 3.h Meta-analysis forest plot for the knee function-KSS 2011 improvement

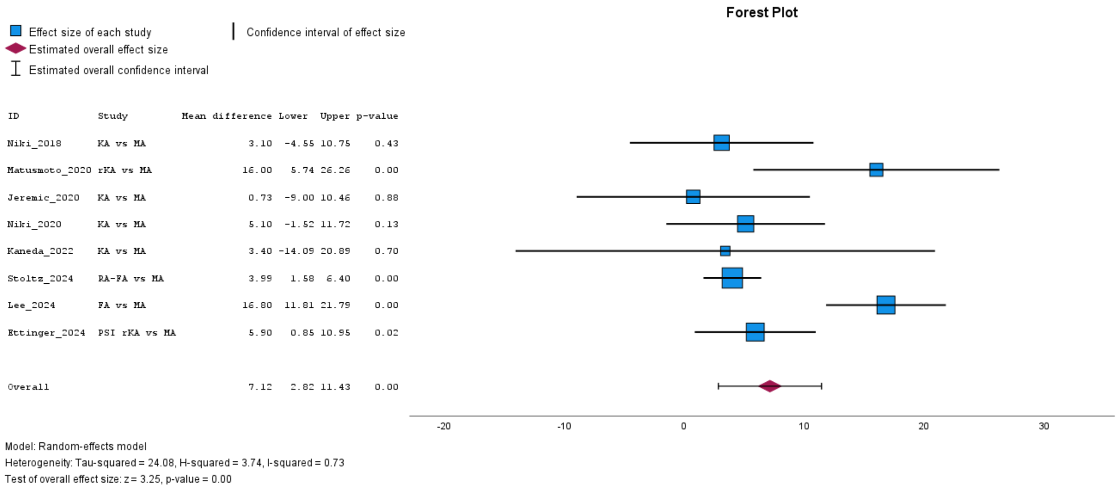

Fig. 3.i Low-to-moderate risk of bias subgroup meta-analysis for the knee function-KSS 2011 improvement

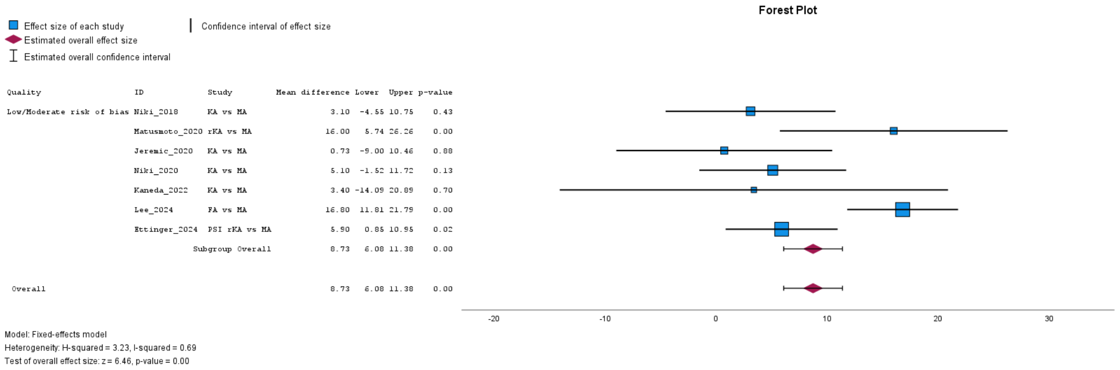

Fig. 4.a Forest plot assessing homogeneity between KOOS-JR and KOOS Symptoms, Pain, and ADL subscales

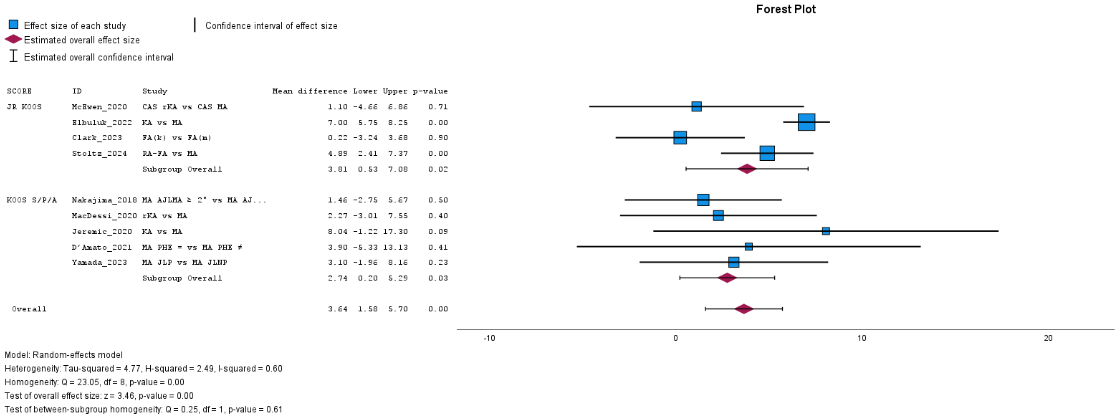

Fig. 4.b Meta-analysis forest plot for the KOOS-JR

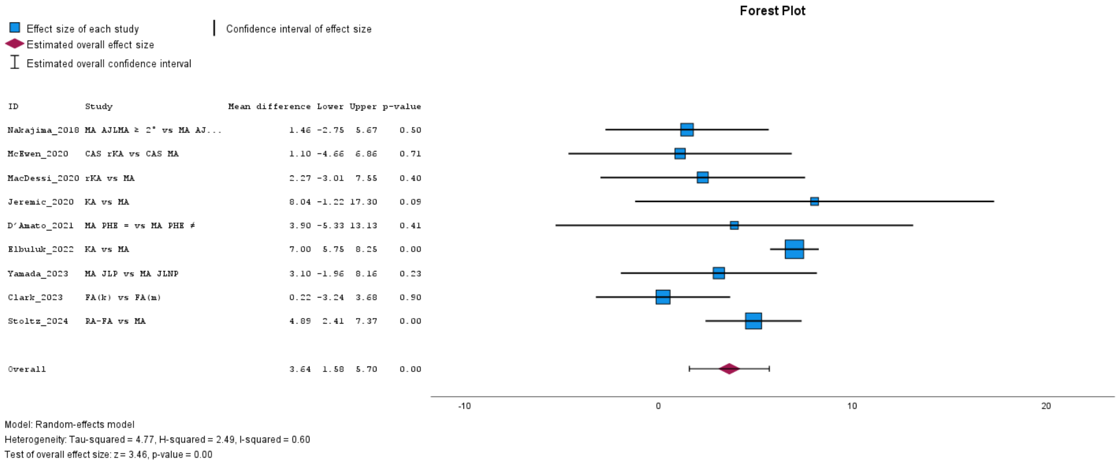

Fig. 4.c Low-to-moderate risk of bias subgroup meta-analysis for the KOOS-JR

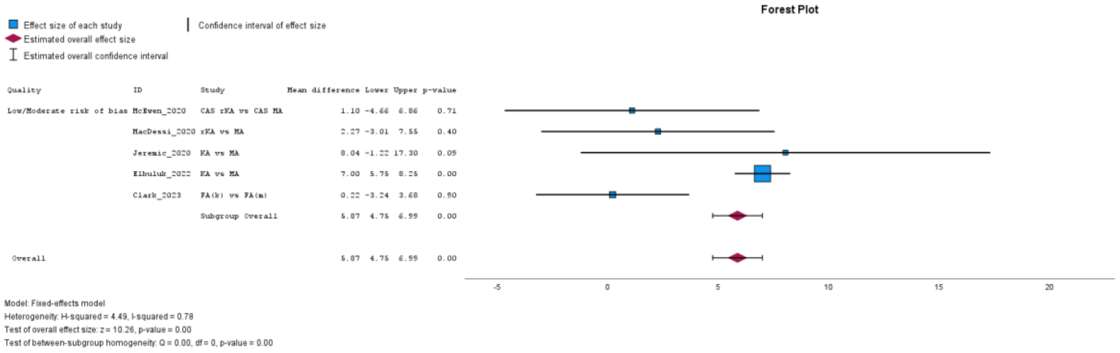

Fig. 4.d Meta-analysis forest plot for the KOOS-JR improvement

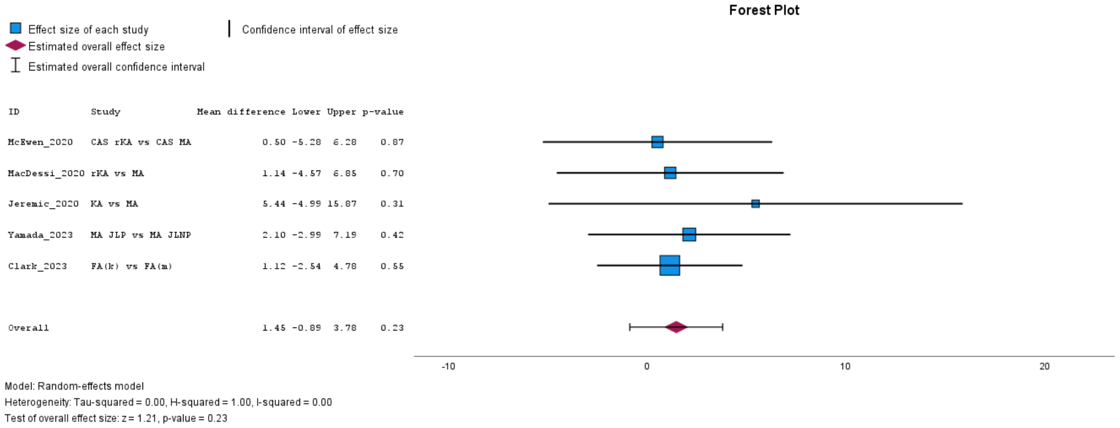

Fig. 4.e Low-to-moderate risk of bias subgroup meta-analysis for the KOOS-JR improvement

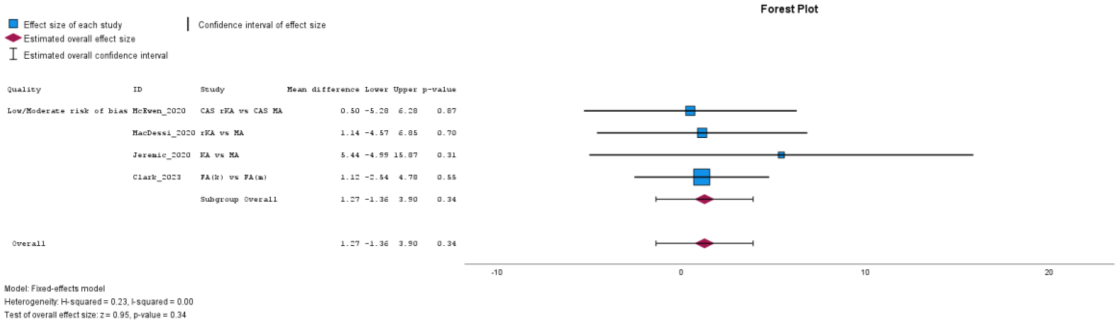

Fig. 5.a Meta-analysis forest plot for the KOOS

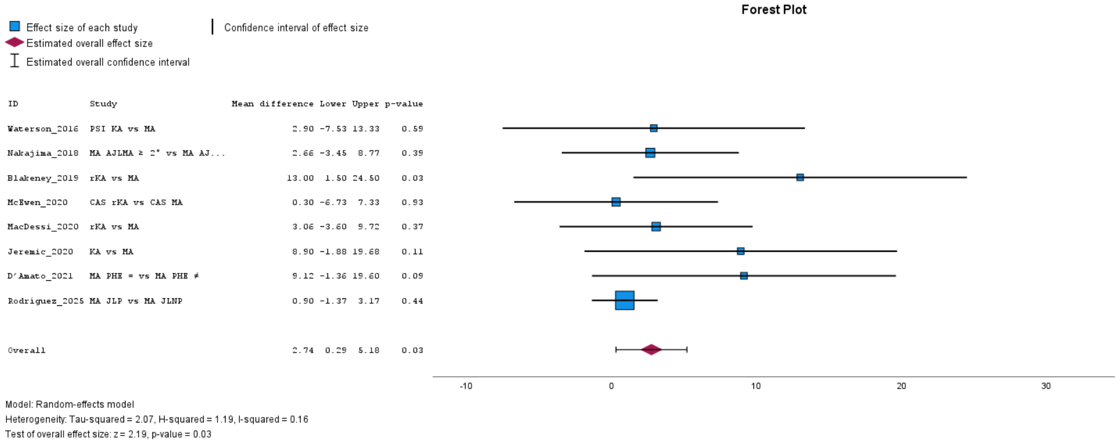

Fig. 5.b Low-to-moderate risk of bias subgroup meta-analysis for the KOOS

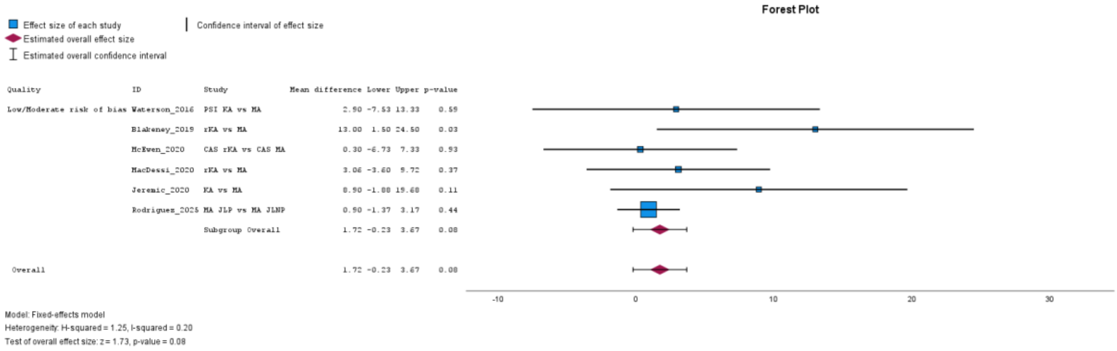

Fig. 5.c Meta-analysis forest plot for the KOOS improvement (all low-to-moderate risk of bias studies)

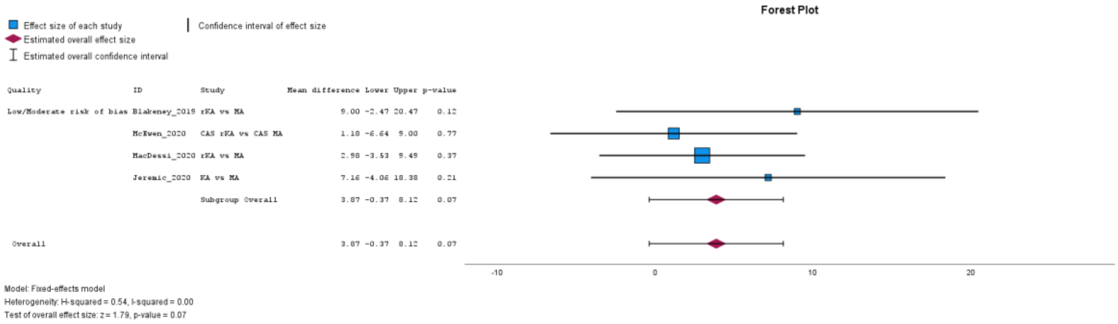

Fig. 6.a Meta-analysis forest plot for the OKS (all low-to-moderate risk of bias studies)

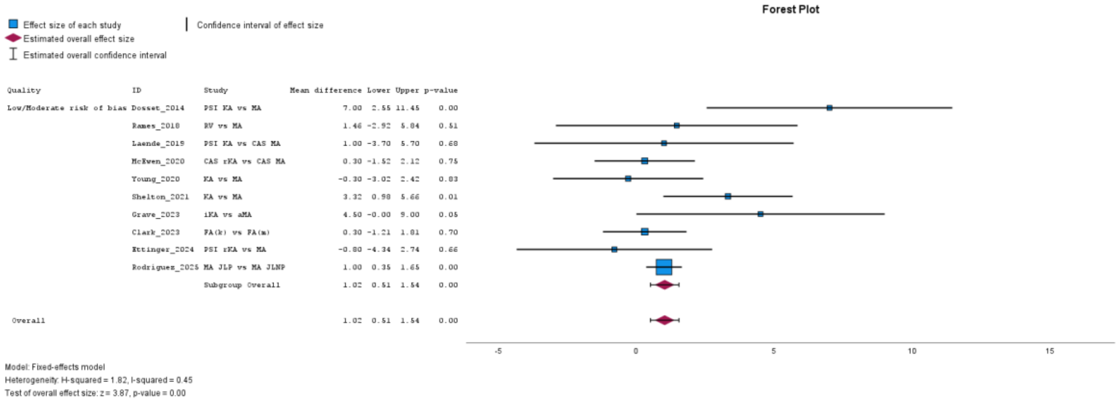

Fig. 6.c Meta-analysis forest plot for the OKS improvement (all low-to-moderate risk of bias studies)

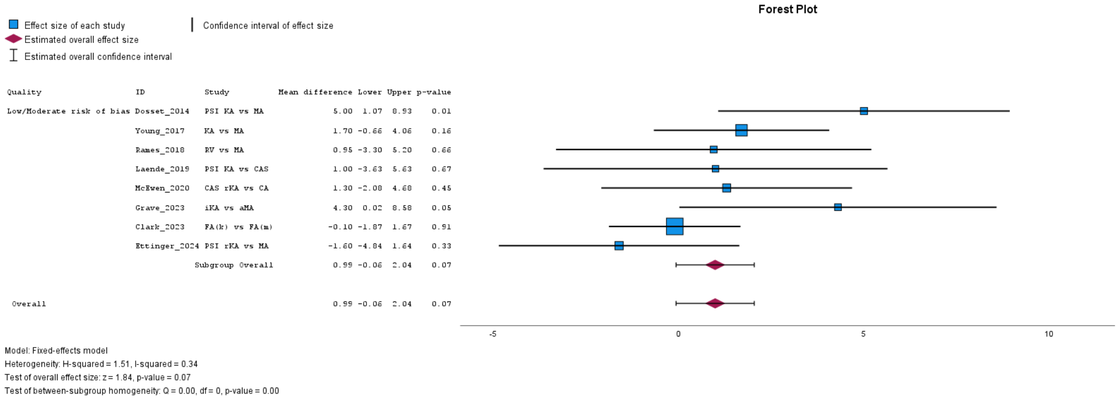

Fig. 7.a Meta-analysis forest plot for the WOMAC

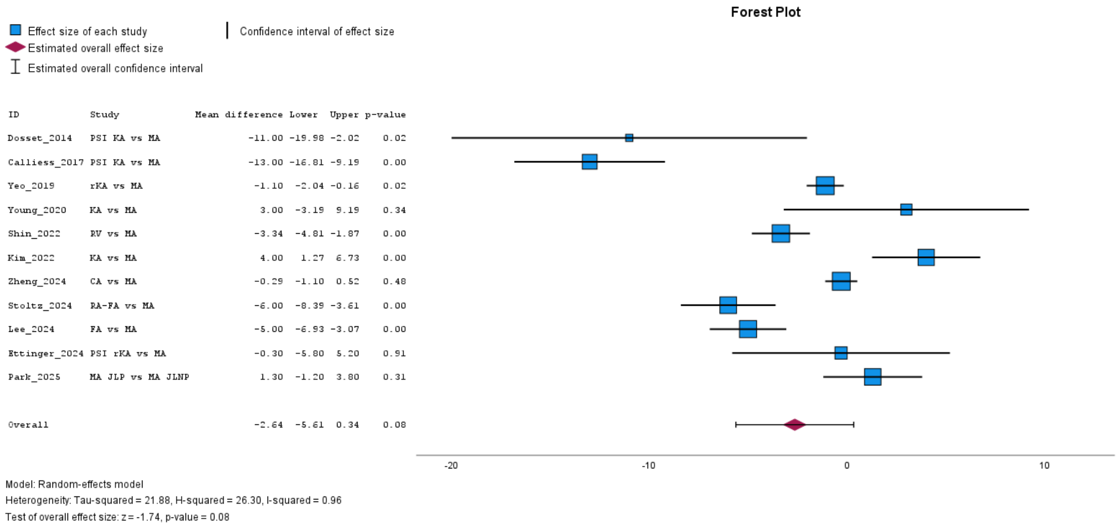

Fig. 7.b Low-to-moderate risk of bias subgroup meta-analysis for the WOMAC

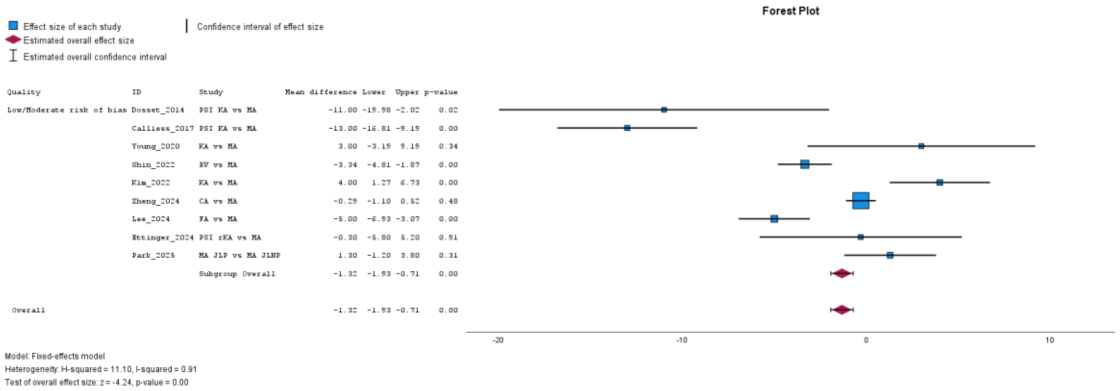

Fig. 7.c Meta-analysis forest plot for the WOMAC improvement

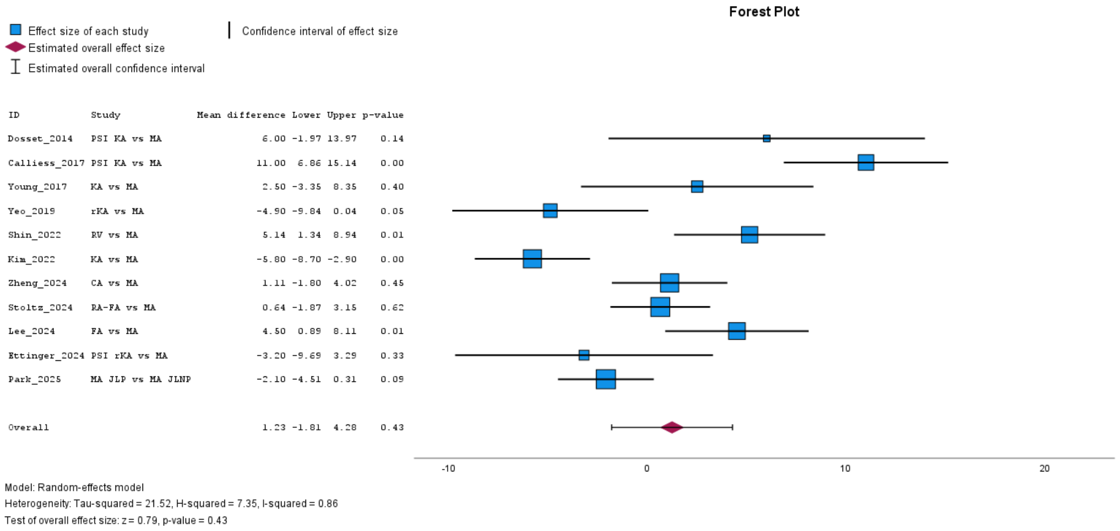

Fig. 7.d Low-to-moderate risk of bias subgroup meta-analysis for the WOMAC improvement

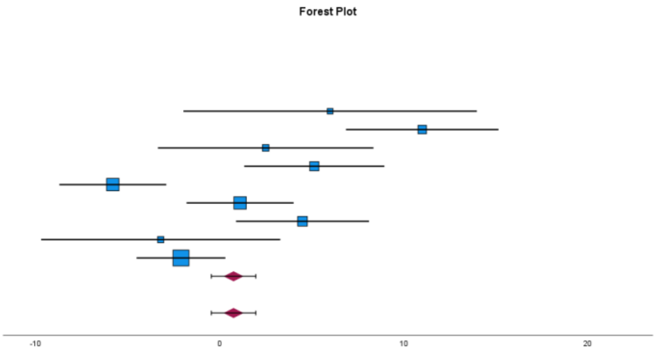

Effect size of each study | Confidence interval of effect size
Estimated overall effect size
Estimated overall confidence interval

Quality	ID	Study	Mean difference	Lower	Upper	p-value
Low/Moderate risk of bias	Dosset_2014	PSI KA vs MA	6.00	-1.97	13.97	0.14
	Calliess_2017	PSI KA vs MA	11.00	6.86	15.14	0.00
	Young_2017	KA vs MA	2.50	-3.35	8.35	0.40
	Shin_2022	RV vs MA	5.14	1.34	8.94	0.01
	Kim_2022	KA vs MA	-5.80	-8.70	-2.90	0.00
	Zheng_2024	CA vs MA	1.11	-1.80	4.02	0.45
	Lee_2024	FA vs MA	4.50	0.89	8.11	0.01
	Ettinger_2024	PSI rKA vs MA	-3.20	-9.69	3.29	0.33
	Park_2025	MA JLP vs MA JLNP	-2.10	-4.51	0.31	0.09
		Subgroup Overall	0.76	-0.46	1.97	0.22
Overall			0.76	-0.46	1.97	0.22

Model: Fixed-effects model
Heterogeneity: H-squared = 7.65, I-squared = 0.87
Test of overall effect size: z = 1.22, p-value = 0.22

Fig. 8.a Meta-analysis forest plot for the ROM

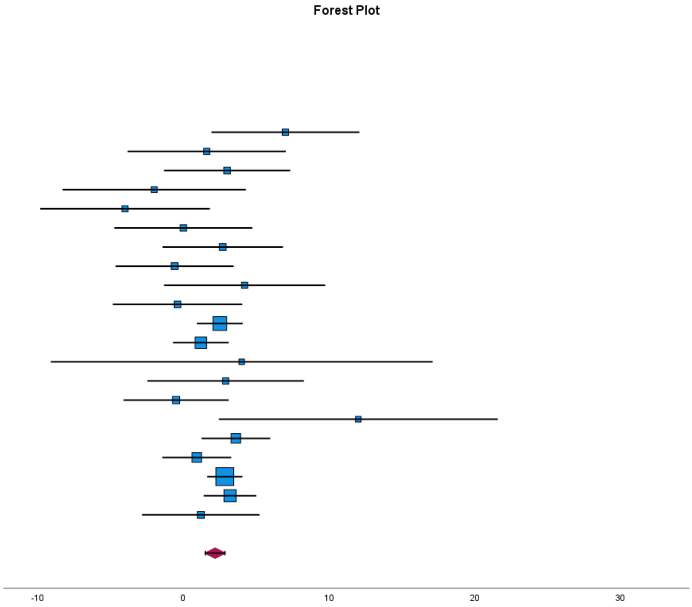

Effect size of each study | Confidence interval of effect size
Estimated overall effect size
Estimated overall confidence interval

ID	Study	Mean difference	Lower	Upper	p-value
Dosset_2014	PSI KA vs MA	7.00	1.93	12.07	0.01
Waterson_2016	PSI KA vs MA	1.60	-3.83	7.03	0.56
Young_2017	KA vs MA	3.00	-1.33	7.33	0.17
Niki_2018	KA vs MA	-2.00	-8.28	4.28	0.53
Yeo_2019	rKA vs MA	-4.00	-9.82	1.82	0.18
McEwen_2020	CAS rKA vs CAS MA	0.00	-4.73	4.73	1.00
Matusmoto_2020	rKA vs MA	2.70	-1.43	6.83	0.20
Niki_2020	KA vs MA	-0.60	-4.65	3.45	0.77
Yaron_2021	KA vs MA	4.20	-1.33	9.73	0.14
Calek_2022	MA JLP vs MA JLNP	-0.40	-4.84	4.04	0.86
Kim_2022	KA vs MA	2.50	0.93	4.07	0.00
Sappey-Marinier_2022	MA JLP vs MA JLNP	1.20	-0.71	3.11	0.22
Elbuluk_2022	KA vs MA	4.00	-9.09	17.09	0.55
Parratte_2023	AFIP vs aMA	2.90	-2.47	8.27	0.29
Yamada_2023	MA JLP vs MA JLNP	-0.50	-4.11	3.11	0.79
Grave_2023	iKA vs aMA	12.00	2.43	21.57	0.01
Clark_2023	FA(k) vs FA(m)	3.60	1.24	5.96	0.00
Zheng_2024	CA vs MA	0.92	-1.44	3.28	0.45
Stoltz_2024	RA-FA vs MA	2.84	1.63	4.05	0.00
Park_2025	MA JLP vs MA JLNP	3.20	1.40	5.00	0.00
Nakagawa_2025	AA vs MA	1.20	-2.83	5.23	0.56
Overall		2.18	1.50	2.85	0.00

Model: Random-effects model
Heterogeneity: Tau-squared = 0.17, H-squared = 1.08, I-squared = 0.07
Test of overall effect size: z = 6.33, p-value = 0.00

Fig. 8.b Low risk of bias subgroup meta-analysis for the ROM

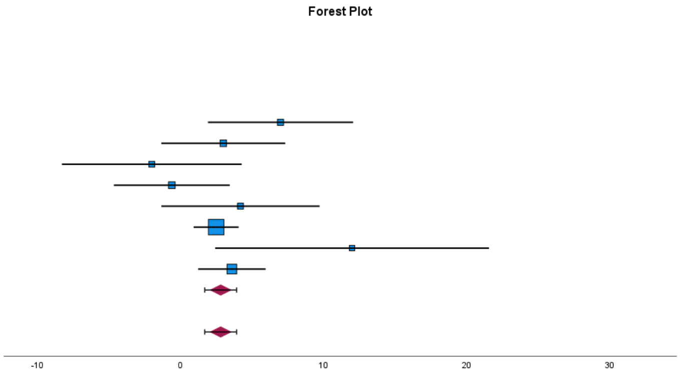

Effect size of each study | Confidence interval of effect size
Estimated overall effect size
Estimated overall confidence interval

Quality	ID	Study	Mean difference	Lower	Upper	p-value
Low risk of bias	Dosset_2014	PSI KA vs MA	7.00	1.93	12.07	0.01
	Young_2017	KA vs MA	3.00	-1.33	7.33	0.17
	Niki_2018	KA vs MA	-2.00	-8.28	4.28	0.53
	Niki_2020	KA vs MA	-0.60	-4.65	3.45	0.77
	Yaron_2021	KA vs MA	4.20	-1.33	9.73	0.14
	Kim_2022	KA vs MA	2.50	0.93	4.07	0.00
	Grave_2023	iKA vs aMA	12.00	2.43	21.57	0.01
	Clark_2023	FA(k) vs FA(m)	3.60	1.24	5.96	0.00
		Subgroup Overall	2.82	1.70	3.93	0.00
Overall			2.82	1.70	3.93	0.00

Model: Fixed-effects model
Heterogeneity: H-squared = 1.71, I-squared = 0.42
Test of overall effect size: z = 4.96, p-value = 0.00

Fig. 8.c Meta-analysis forest plot for the ROM improvement

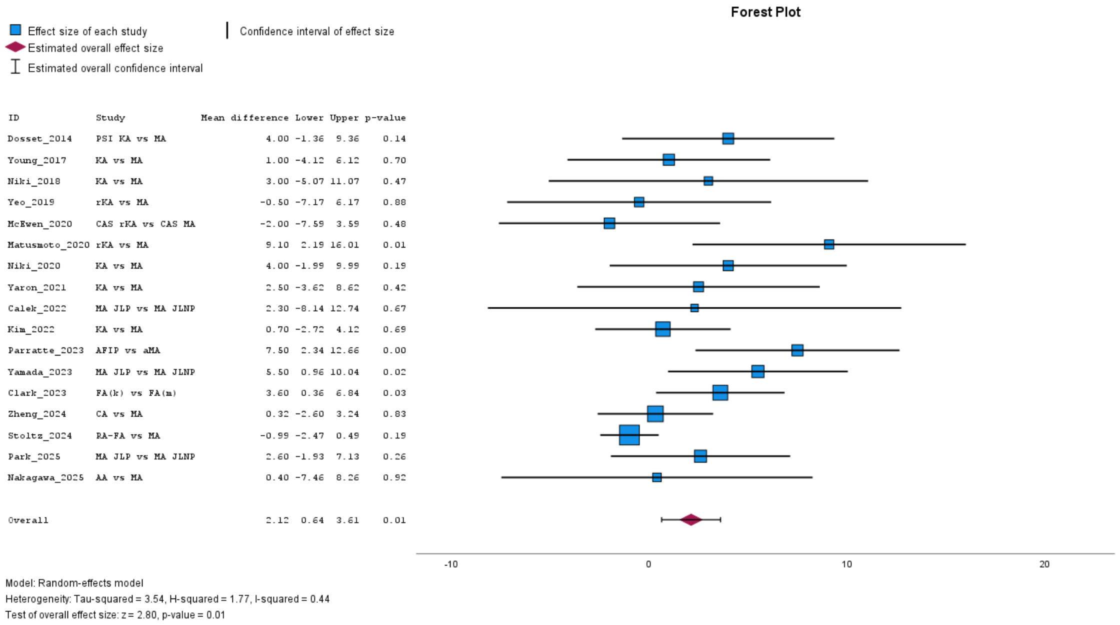

Fig. 8.d Low risk of bias subgroup meta-analysis for the ROM improvement

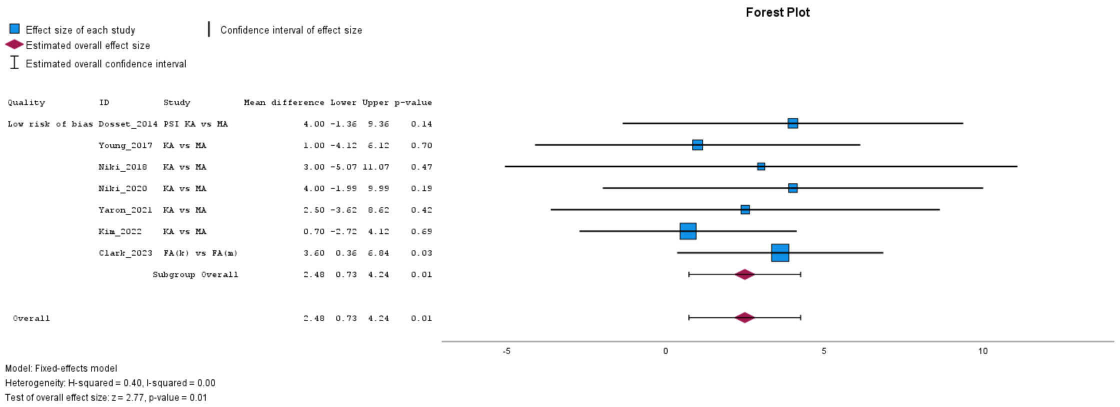

Supplement: Supplementary file 1 — Supporting information. [file JEO2-12-e70458-s001.pdf]
